# The dishwasher rubber seal acts as a reservoir of bacteria in the home environment

**DOI:** 10.1186/s12866-019-1674-5

**Published:** 2019-12-19

**Authors:** Jerneja Zupančič, Martina Turk, Miha Črnigoj, Jerneja Ambrožič Avguštin, Nina Gunde-Cimerman

**Affiliations:** 1 Department of Biology, Biotechnical Faculty, University of Ljubljana, Ljubljana, Slovenia; 2 Centre of Excellence for Integrated Approaches in Chemistry and Biology of Proteins (CIPKeBiP), Ljubljana, Slovenia

**Keywords:** Kitchen, Dishwasher, Bacteria, Antibiotic resistance, Tap water

## Abstract

**Background:**

In modern lifestyles, people make their everyday tasks easier by using household appliances, for example dishwashers. Previous studies showed massive contamination of dishwasher rubber seals with fungi, thus bacterial community, able to survive under harsh conditions, remain undetermined.

**Methods:**

Bacteria that colonise the extreme environment of household dishwasher rubber seals were investigated using cultivation-dependent and metagenomic approaches. All bacterial isolates were tested for resistance to seven selected antibiotics. Same time bacterial diversity of tap water, connected to the dishwashers was investigated.

**Results:**

All 30 dishwashers investigated were colonised by various bacteria. Cultivation approaches resulted in 632 bacterial isolates in total, belonging to four phyla, eight classes, 40 genera and 74 species. The majority were Gram-positive, as solely Firmicutes (dominated by the *Bacillus cereus* group) and Actinobacteria. Gammaproteobacteria were primarily represented by *Stenotrophomonas maltophilia*, *Pseudomonas aeruginosa* and *Escherichia coli*. Metagenomic assessment of the bacterial biodiversity of the dishwasher rubber seals confirmed the predominance of Gram-positive bacteria, as primarily Actinobacteria, followed by Proteobacteria dominated by Gammaproteobacteria, and by pathogenic species such as *Escherichia* sp., *Acinetobacter baumannii*, *Pseudomonas* sp., *Stenotrophomonas maltophilia*, and *Enterobacter* sp.. Metagenomic assessment of bacterial biodiversity in the tap water connected to dishwashers revealed predominance of Gram-negative bacteria, in particular Proteobacteria, mainly represented by *Tepidimonas* sp.. Actinobacteria showed low numbers while no Firmicutes were detected in the tap water. The bacterial diversity of tap water was also lower, 23 genera compared to 39 genera on dishwasher rubber seals. Only 13 out of 49 genera identified by metagenomics approach was found in both environments, of those *Gordonia* was enriched while half of 13 genera were depleted in dishwashers compared to tap water.

**Conclusions:**

These data indicate that colonisation of dishwasher rubber seals probably depends primarily on the bacterial input from the dirty vessels, and much less on the bacteria in the tap water. Based on the antibiotic resistance data, the dishwasher rubber seal bacterial isolates do not represent a serious threat for the spread of antibiotic resistance into the household environment. Nevertheless dishwashers cannot be ignored as potential sources of human infections, in particular for immuno-compromised individuals.

## Background

Humans have modified the environment in which they live throughout their entire history. As a consequence, indoor dwellings have become increasingly isolated from the outdoor environment [55], and subject to increased sanitation due to intensive use of chemicals and disinfectants. As nowadays we spend most of our time indoors, conditions in these indoor environments are increasingly influencing our health [14, 28, 36, 50].

On the other hand, stress tolerance and the great adaptability of some microorganisms means that they can inhabit novel habitats that have previously been considered as hostile to abundant microbial growth [24]. Surveys of indoor habitats have, for example, uncovered a surprising diversity of polyextremotolerant opportunistic and pathogenic bacteria [17, 18] and fungi [27].

In these indoor habitats, the microbes are exposed to conditions that are similar to those encountered in nature, but are nevertheless different in important details. Kitchens are characterised by the presence of running water, food remains, frequent contact with humans, and intense use of chemicals and disinfectants [20, 43], and can be heavily colonised by bacteria and other microbes [19, 20, 47, 48, 57, 61]. These adapted microorganisms invade not only different surfaces and wet environments in the kitchen [20], but also within household appliances.

Domestic dishwashers as an environment were not considered to pose any threat to humans until Zalar et al. [70] revealed heavy contamination of dishwasher rubber seals with selected opportunistic pathogenic fungi [13, 23, 71]. This fungal contamination was not limited to the rubber seals, but was spread over the entire interiors of the dishwashers, which provided an environment that influenced the microbiota throughout the kitchen [71].

Besides fungi, also bacteria can contaminate dishwashers as revealed in limited studies focusing on dishwasher bacterial contamination [52, 53, 72]. Surprisingly, the prevous studies performed on bacteria in dishwashers were focused on dishwasher sanitising performance, in terms of the determination of the survival of certain selected pathogenic bacterial species during the washing cycle and on the washed eating utensils [40, 46, 62].

The present study was thus focused on the diversity of the bacterial communities that might be found to colonise dishwasher rubber seals, with the sampling of 30 randomly picked household dishwashers, and in the tap water systems connected to them, using both cultivation-dependent and metagenomic approaches. As a significant number of people are affected by infections each year that are caused by antibiotic-resistant bacteria, which very often cause severe complications or death, all of the bacterial isolates obtained from these dishwasher seals were tested for resistance to a selection of antibiotics.

## Results

### Dishwasher rubber seals are populated with diverse bacterial communities dominated by gram-positive bacteria

All 30 sampled residential dishwasher rubber seals were colonised by bacteria. In total, 632 bacterial isolates were obtained that belonged to four phyla, eight classes, 40 genera and 74 species (Table 1). On average, the dishwashers were contaminated with four to eight different bacterial species, while three of the 30 dishwashers showed higher cultivable bacterial diversity, as 15, 17 and 22 different bacterial species were isolated from three separate dishwasher rubber seals (Additional file 1: Table S1).

**Table 1:** Bacterial species found across the 30 sampled dishwasher rubber seals

Phylum	Class	Genus	Species	Dishwasher																													
				1	2	3	4	5	6	7	8	9	10	11	12	13	14	15	19	20	21	22	23	24	25	27	28	29	30	31	32	33	34
Proteobacteria	*Alphaproteobacteria*	*Rhizobium*	*radiobacter*																					x				x					
		*Ochrobactrum*	sp.		x								x																				
		*Brevundimonas*	*diminuta*																					x									
		*Roseomonas*	*cervicalis*						x															x									
		*Sphingomonas*	*mucosissima*																					x									
			*paucimobilis*																									x	x				
	*Betaproteobacteria*	*Achromobacter*	*insolitus*										x																				
		*Comamonas*	*aquatica*						x																								
	*Gammaproteobacteria*	*Acinetobacter*	*calcoaceticus*		x				x							x																	
			*junii*				x																										
			*ursingii*																	x													
		*Acinetobacter*	sp.						x										x	x													
		*Pseudomonas*	*aeruginosa*	x	x											x			x					x				x					
			*pseudoalcaligenes*	x							x											x	x					x					
			*stutzeri*								x														x								
		*Pseudomonas*	sp.				x		x													x		x									
		*Cronobacter*	*sakazakii*													x										x							
		*Enterobacter*	sp.		x		x		x						x																		
		*Escherichia*	sp.	x					x				x			x						x			x								
		*Escherichia*	*hermannii*													x																	
		*Klebsiella*	*oxytoca*	x	x								x																				
			*pneumoniae*		x				x							x				x													
		*Pantoea*	*agglomerans*													x																	
		*Raoultella*	*ornithinolytica*		x			x							x																		
		*Stenotrophomonas*	*maltophilia*	x	x			x	x		x		x							x			x	x	x								
Firmicutes	*Bacilli*	*Bacillus*	*amyloliquefaciens*														x								x				x				
			*cereus* group	x	x	x	x	x	x		x	x	x	x				x	x	x	x	x		x		x	x	x	x	x	x	x	x
			*circulans*																	x													
			*firmus*									x																					
			*flexus*			x			x	x		x	x	x					x	x	x		x	x			x			x			x
			*horneckiae*					x				x																					
			*licheniformis*										x																				
			*pumilus*									x		x					x	x								x	x				
			*safensis*						x										x									x					
			*subtilis* group			x	x	x	x		x			x							x					x	x	x	x	x			x
		*Bacillus*	sp.			x	x		x			x	x	x		x			x	x	x		x			x		x			x		
		*Brevibacillus*	*parabrevis*				x						x																				
		*Paenibacillus*	sp.	x		x	x	x				x								x						x	x		x	x	x	x	
		*Staphylococcus*	*pasteuri*										x																				
			*saprophyticus*															x							x			x	x				
			*succinus*																								x						
		*Staphylococcus*	sp.				x		x																			x					
		*Kurthia*	*gibsonii*						x						x							x											
		*Lysinibacillus*	*fusiformis*					x					x							x									x	x	x		
		*Exiguobacterium*	sp.				x		x				x		x							x	x		x			x					x
		*Aerococcus*	*viridans*															x				x											
		*Aerococcus*	sp.															x				x											
		*Enterococcus*	*casseliflavus*	x	x				x											x	x	x		x	x			x					
			*faecium*																	x		x											
	*Clostridia*	*Clostridium*	*xylanolyticum*										x																				
Bacterioidetes	*Flavobacteria*	*Chryseobacterium*	sp.		x		x		x				x																				
		*Flavobacterium*	*lindanitolerans*																						x								
	*Sphingobacteria*	*Sphingobacterium*	*multivorum*																										x				
Actinobacteria	*Actinobacteria*	*Aeromicrobium*	sp.						x																								
		*Gordonia*	*bronchialis*															x															
			*paraffinivorans*						x																x					x			
			*polyisoprenivorans*															x															
			*terrae*																							x							
		*Brachybacterium*	sp.																									x					
		*Brevibacterium*	*casei*															x				x			x	x						x	
			*sanguinus*																													x	
		*Corynebacterium*	sp.																				x										
		*Kocuria*	*kristinae*		x																												
			*rhizophila*																													x	
		*Microbacterium*	*aurum*																											x			
			*lacticum*							x																							
			*oxydans*					x								x																	
		*Microbacterium*	sp.										x																				
		*Micrococcus*	*luteus*							x			x	x						x			x					x	x	x			x
		*Micrococcus*	sp.						x	x								x					x						x				x
		*Cellulosimicrobium*	*cellulans*																					x	x					x			
		*Naumannella*	*halotolerans*														x																

**Fig. 1:**
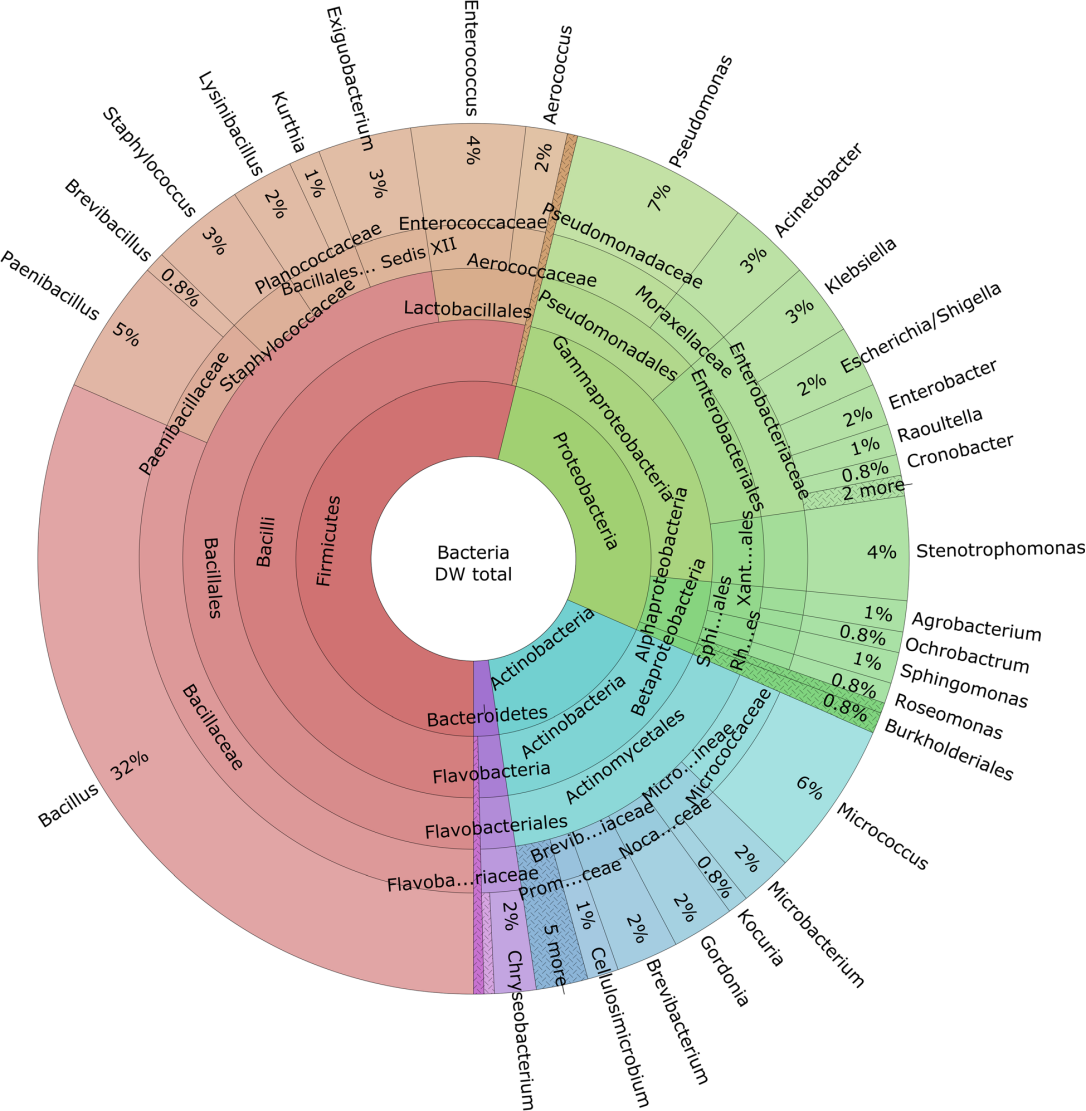
Diversity and abundance of the bacterial species isolated from the swab samples from the 30 residential dishwasher rubber seals. The most represented phylum was Firmicutes (54%), followed by Proteobacteria (28%), Actinobacteria (16%) and Bacteroidetes (2%). The dominant classes were Bacilli (53%), Actinobacteria (16%) and Gammaproteobacteria (23%)

Sixty-five percent (48/74) of the isolated species were Gram-positive, which were represented solely by Firmicutes and Actinobacteria. Class Bacilli represented 50% (24/48) of all Gram-positive isolates. The remaining 35% (26/74) of the isolate species were Gram-negative, and these were most abundantly represented by class Gammaproteobacteria (65%; 17/26) (Table 1). On average, the dominant classes were represented by Bacilli (53%), Actinobacteria (16%) and Gammaproteobacteria (23%) (Fig. 1).

### The *Bacillus cereus* group is the dominant contaminant of the dishwasher rubber seals

Bacilli (Firmicutes), as primarily the *Bacillus cereus* group, the *Bacillus subtilis* group, *Bacillus flexus*, *Bacillus* sp. and *Paenibacillus* sp. were most frequently isolated from the dishwasher rubber seals (Table 1). The overall predominance was for isolates of the *B. cereus* group, which were isolated from 80% (24/30) of the dishwashers sampled. The second most commonly isolated species were *Bacillus* sp. and *B. flexus* (both 47%; 14/30), followed by the *B. subtilis* group and *Paenibacillus* sp. (43%; 13/30). Amongst Actinobacteria, *Micrococcus luteus* was most frequent (30%; 9/30), followed by *Micrococcus* sp. (20%; 6/30) and *Brevibacterium casei* (17%; 5/30). Gammaproteobacteria were primarily represented by *Stenotrophomonas maltophilia* (33%; 10/30), *Pseudomonas aeruginosa* and *Escherichia* sp. (both 20%; 6/30) (Fig. 1).

### Metagenomic assessment of bacterial biodiversity from dishwasher rubber seals confirms predominance of gram-positive bacteria

**Fig. 2:**
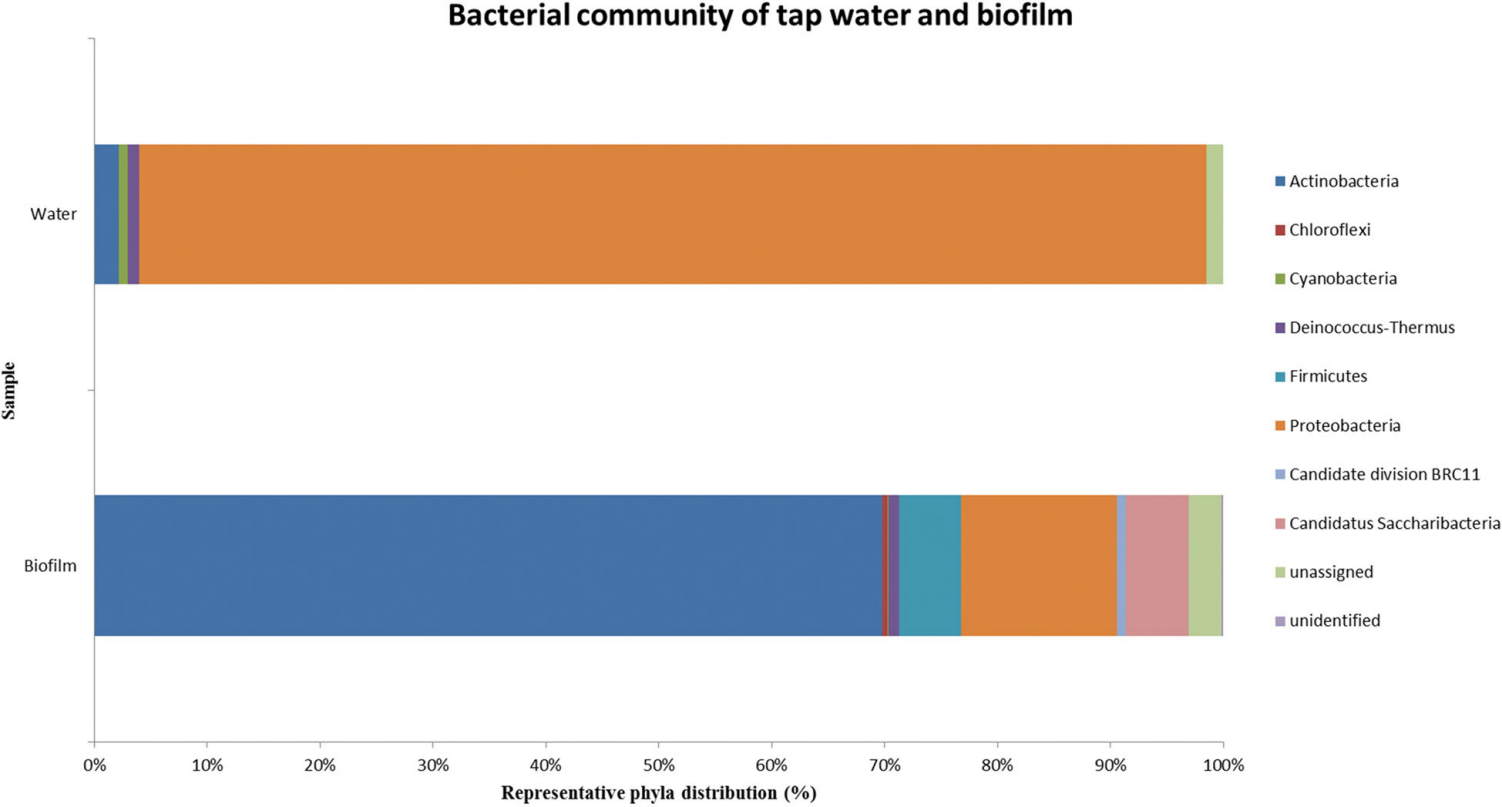
Proportions of the different bacterial phyla from the tap water system and the biofilms from the dishwasher rubber seals. Although the broad composition of bacterial taxa was similar across the different samples, their relative abundances varied, indicating that dishwashers are highly selective environments

To gain further insight into the diversity of the non-cultivable part of the bacterial communities that inhabited these dishwasher rubber seals, pyrosequencing was performed for the 16S rRNA gene from DNA isolated from the biofilms on the dishwasher rubber seals and from the tap water connected to the dishwashers. Analysis of these metagenomic data resulted in 4638 reads assigned to OTUs from the dishwasher biofilms, and 1503 reads assigned to OTUs from the water samples (Fig. 2, Additional file 1: Table S2). The majority of the OTUs from the biofilms were assigned to Gram-positive bacteria (76%), and primarily to phylum Actinobacteria (70%), which was mainly represented by *Gordonia* sp. (66%), followed by 14 other genera. Proteobacteria were the second most common bacterial phylum (14%), with a predominance of Gammaproteobacteria (73%) where opportunistic pathogenic species such as *Escherichia* sp., *Acinetobacter baumannii*, *Pseudomonas* sp., *Stenotrophomonas maltophilia* and *Enterobacter* sp. were detected, together with 3 other genera. Other abundant sequences were affiliated to the phylum Firmicutes (6%), out of which *Exiguobacterium* sp. was the most numerous representative (51%).

### Metagenomic assessment of bacterial biodiversity in the tap water reveals apredominance of gram-negative bacteria

The metagenomics analysis of the tap water samples demonstrated the prevalence of OTUs assigned to Gram-negative bacteria (95%), among which Proteobacteria (95%) in particular were detected (Fig. 2, Additional file 1: Table S2). Betaproteobacteria were the most abundant (62%) among Proteobacteria, but were mainly represented by *Tepidimonas* sp. (92%), followed by 4 other genera. Actinobacteria were present in low numbers (2%, 3 genera) while Firmicutes were not present at all in tap water. Both Alphaproteobacteria and Betaproteobacteria were markedly more present in the tap water (85%; 352/1503 OTUs plus 929/1503 OTUs, respectively) with observed greater diversity for these two taxons as well (10 and 5 genera, respectively), and were hardly found in the dishwasher biofilms (4%; 99/4638 OTUs plus 75/4638 OTUs, respectively). The reverse situation was observed for Gammaproteobacteria, which were present only at 9% in the tap water (139/1503 OTUs) with lower diversity (4 genera), but were abundant and showed greater diversity in the dishwasher biofilms (467/4638OTUs and 8 genera) (Fig. 2). Of 49 genera identified, 10 were detected only in tab water and 26 only on dishwasher rubber seals. Additional file 1: Table S2 presents all of the reads obtained.

### Bacterial diversity on dishwasher rubber seals is mostly influenced by water hardness and washing temperature

**Fig. 3:**
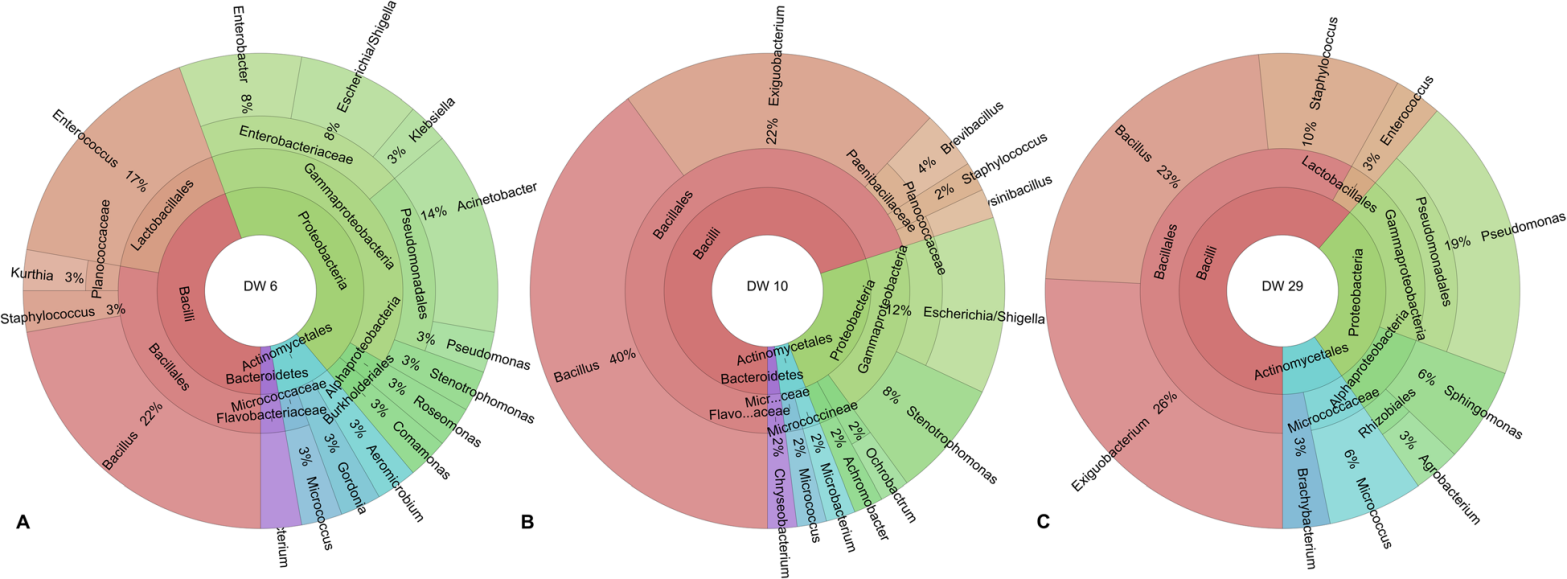
Comparisons of the bacterial species diversity on the rubber seals of the three sampled dishwashers that showed the highest cultivation-dependent bacterial diversity, which were supplied with hard tap water. Dishwasher 6 (**a**) and dishwasher 10 (**b**) were both supplied with moderately hard tap water (1.6 mmol/L CaCO_3_), and dishwasher 29 (**c**) was supplied with hard tap water (2.0 mmol/L CaCO_3_). Different colors convey bacterial classes e.g. Bacilli are represented in red, Proteobacteria are represented in green, Actinomycetes are represented in blue and Bacteroidetes are represented in purple colour

These randomly selected dishwashers were characterised according to the type of water supply (from hard to soft), age (years since purchased), frequency of use (times per week), cleaning (method) and temperature of washing (Table 2; for full details, see Additional file 1: Table S1). The highest cultivation-dependent bacterial diversity for the rubber seals (15–22 different bacterial species) was detected in dishwashers connected to hard or moderately hard tap water (1.5–2.0 mmol/L CaCO_3_) (Fig. 3). A closer look at isolates from these three dishwashers with the highest bacterial diversity (Table 1) showed that the most frequent species on the rubber seals was *Exiguobacterium* sp., which represented 26% of all of the isolated species (dishwashers 10, 29), and *Enterococcus casseliflavus*, which represented 17% of all of the isolated species (dishwasher 6). For the rubber seals, dishwashers 10 and 29 had 61 and 70% Bacilli, 24 and 29% Proteobacteria, and 4 and 10% Actinobacteria, respectively, while dishwasher 6 had equal levels of Bacilli and Proteobacteria (44%), with 8% Actinobacteria. *Escherichia* sp. was present on the rubber seals of both dishwashers 6 and 10 (8 and 12% of all isolates, respectively), but not of dishwasher 29 (Fig. 3).

**Table 2:** Characteristics of the dishwashers in relation to the mean numbers of different bacterial species isolated

Characteristic	Specific	Mean bacterial species per dishwasher
Water hardness (mmol/L CaCO_3_)	Hard (2.0)	7.8
	Moderately hard (1.6)	9.6
	Slightly hard (1.0)	9.0
	Soft (0.5)	4.5
Type of cleaning	None	8.9
	Chemical	8.5
	Mechanical	11.0
Temperature of washing (°C)	50	10.3
	60	9.1
	65	8.9
	70	2.0
Frequency of use (per week)	< 7	9.2
	7	7.9
	8–14	11.0
Age of dishwasher (years)	0.5–1.0	10.9
	1.1–2.0	9.8
	2.1–3.0	7.8
	3.1–6.0	8.3
	6.1–8.0	7.7

In comparison, for the rubber seals of the two dishwashers connected to soft tap water (0.5 mmol/L CaCO_3_), only up to six different bacterial species were detected. These isolated species belonged to Firmicutes (*B. cereus* group, *Paenibacillus* sp.) and Actinobacteria (*Brevibacterium casei*, *Brevibacterium sanguinus*, *Kocuria rhizophila*) (Table 1). Proteobacteria (which includes *Escherichia* sp. and *P. aeruginosa*) and Bacteroidetes were not detected on the rubber seals of these dishwashers, whereas *K. rhizophila* was isolated only from the rubber seals of dishwashers connected to soft tap water.

Bacterial diversity was influenced also by the frequency of use and the age of the dishwashers (i.e., years from purchase). More frequent use and up to 1 year of operation time was associated with higher bacterial diversity here, while the number of isolated species decreased with the age of the dishwashers, reaching the lowest levels for 6–8-year-old dishwashers (Table 2).

With a temperature of washing of approximately 50 °C, this was associated with the highest diversity of isolated bacteria (as a mean of 10.3 different bacterial species per rubber seal), with the higher temperatures indicating lower numbers of species detected (Table 2).

No differences in the cultivable bacterial diversity were observed in relation to the method of cleaning of the dishwashers (Table 2).

### Selected gram-positive bacteria can contaminate washed dishes

The presence and diversity of bacteria on the dishes and cutlery immediately after the end of the washing process were also investigated. All of the isolates obtained (15) were Gram-positive species that belong to the phyla Firmicutes and Actinobacteria. The highest bacterial diversity was observed after the sampling of some plastic items, whereas this was lower for the metal and ceramic objects. For example, *Gordonia paraffinivorans*, *Brachybacterium nesterenkovii*, *Micrococcus* sp., and *M. luteus* were isolated from a plastic meat-cutting board, *Bacillus flexus*, *Bacillus marisflavi*, and *M. luteus* from a glass lid, and *M. luteus* from a ceramic plate and from metal cutlery.

### Bacterial communities that contaminate dishwasher rubber seals do not represent a serious threat for the spread of antibiotic resistance

From 632 isolates tested for antibiotic resistance against the seven selected antibiotics, the majority (48%) was represented by Firmicutes from the order Bacilliales (*Bacillaceae*, *Paenibacillaceae*, *Planococcaceae*, *Staphylococcaceae*) and are presented in Additional file 1: Table S3. This group showed relatively low levels of antibiotic resistance, with the highest seen for the third generation cephalosporins (cefotaxime 57%, ceftazidime 70%), while the resistance against the other screened antibiotics was 10 to 12%, or lower (for full details, see Additional file 1: Table S3).

For the order of the Firmicutes, Lactobacillales (e.g., *Enterococccaceae*), there was high antibiotic resistance against cefotaxime, ceftazidime, ertapenem and ciprofloxacin (90% of isolates). The order of Actinomycetales showed slightly elevated resistance against ceftazidime (60%) and cefotaxime (81%).

Among the Proteobacteria, Pseudomonadales showed the highest levels of resistance against cefotaxime (80%) and ertapenem (51%). The order of Enterobacteriales did not show any particularly resistance, as all of these were < 8%, with the exception of resistance to imipenem (25%).

Isolates belonging to the orders of Xanthomonadales (Proteobacteria) and Flavobacteriales (Bacterioidetes) were not as numerous as for the previous groups; however, they showed relatively high levels of antibiotic resistance. In Xanthomonadales, the resistance against all used antibiotics except tetracycline was between 76 and 100%, and was indeed mainly > 90%, while in Flavobacteriales all of the resistance was between 80 and 90%, except for ceftazidime (60%).

The groups of Sphingomonadales, Rhizobiales and Rhodospirillales showed higher resistance against cefotaxime and ceftazidime (both at 86%).

## Discussion

Over the last two decades there have been several reports of home-related microbial infections [6, 8, 37, 58, 60]. Out of all of the indoor locations, bathrooms [18, 19, 35] and kitchens [20, 47, 48, 57, 61] are among the most heavily colonised by opportunistic pathogenic bacteria, both in terms of abundance and diversity. Although the spread of most food-related pathogenic bacteria (e.g., *Campylobacter*, *Salmonella*, *Listeria*) [3, 29, 42] can be minimised using correct hygiene practices and disinfectants [10, 11, 54, 59], advances in technology and increasingly inhospitable indoor habitats to microbes have driven the selection of different and more stress-resistant species.

Some studies have reported that there is a link between the metabolism of phenols and hydrocarbons and the microbial tendency to infect the central nervous system [51]. Repeated cycles of thermal stress in house appliances select for thermotolerant, opportunistic pathogens [24, 25]. Opportunistic pathogenic bacteria [56, 69] and fungi (Novak [1]) contaminate washing machines. Bacteraemia outbreaks of *B. cereus* have been reported for hospitals using linen that was washed in contaminated washing machines [56], and for *Gordonia bronchialis* after laundering of surgical scrubs in domestic washing machines [69].

Although dishwashers are also heavily contaminated with selected opportunistic pathogenic fungi [13, 23, 70, 71], so far there is one report on the diversity and characteristics of the bacterial contaminants in the mixed bacterial-fungal biofilms that can colonise dishwasher rubber seals [72].

Comparisons between the non-cultivable and cultivable bacterial communities that have been isolated from dishwashers have shown the differences in their structures. The predominance of the Firmicutes (54%, 10 genera), followed by Proteobacteria (28%, 16 genera) and Actinobacteria (16%, 10 genera), has been reported among cultivable microorganisms. Analysis of metagenomic data has provided a different picture, with the dominance of Actinobacteria (70%, 15 genera), followed by Proteobacteria (14%, 21 genera), a small percentage of Firmicutes (6%, 2 genera) and some candidate phyla. One of the reasons for the discrepancy is probably the selection of the chosen synthetic microbial media and the cultivation conditions, which favoured the isolation of Firmicutes, and primarily the genus *Bacillus*.

Plumbing systems that supply water to household dishwashers represent the most probable route of contamination of appliances with fungi [71]. Therefore, 30 tap water samples from the kitchens with dishwashers were analysed for the presence of bacteria using a metagenomic approach. A prevalence of Gram-negative bacteria was shown here, and in particular of Proteobacteria, with high prevalence of *Tepidimonas* sp. (Betaproteobacteria), a very low number of Actinobacteria and no Firmicutes. Compared to the biofilms on dishwasher rubber seals the bacterial diversity of tap water was also lower, 23 genera compared to 39 genera on rubber seals. The greatest difference in diversity was observed for Actinobacteria (3 genera in tap water versus 15 genera on dishwasher rubber seals). Only 13 out of 49 genera identified by metagenomics approach was found in both environments, of those *Gordonia* was enriched while half of 13 genera were reduced in dishwashers. This piece of information together with the fact that the microbial communities in these dishwasher rubber seal biofilms were dominated by completely different Gram-positive bacteria show that we can probably rule out the tap water as the main route for introduction of bacteria into these dishwashers, in contrast to what was observed in fungi [71]. Therefore, dirty vessels probably represent the major vehicle of bacterial transfer into these dishwashers.

Close contact of different microbes in well-established microbial biofilms that cover the dishwasher rubber seals can facilitate the spread of antibiotic resistance amongst these, and thus we characterised all of these bacterial isolates in relation to seven selected antibiotics. Among the isolated strains in Firmicutes, *B. amyloliquefaciens* from one dishwasher, together with *B. pumilus* and *B. subtilis,* were resistant to cephalosporins. *Bacillus horneckiae* showed resistance to carbapenem antibiotics (imipenem, ertapenem) and some *B. pumilus* isolates were also resistant to ciprofloxacin and ertapenem. This is in contrast with literature reports that have indicated that species from the genus *Bacillus* are usually susceptible to imipenem, ciprofloxacin and tetracycline, and except for *B. cereus* (which produces a broad spectrum β-lactamase), also to cephalosporins (cefotaxime, ceftazidime) and penicillins [68]. According to the literature data, *Paenibacillus* species are usually susceptible to all of the antibiotics that were used in the present study [68], while these isolates here were resistant to one up to three of the antibiotics tested, with the exception of tetracycline. Although *Staphylococcus saprophyticus*, the second most common pathogen identified in urinary tract infections, is a relatively susceptible organism [30], the isolates in the present study were resistant to the cephalosporins tested.

Among the isolated *Exiguobacterium* sp. strains, only one isolate showed multiple resistance to the antibiotics tested. The genus *Enterococcus* (Lactobacillales) includes some of the most important nosocomial multidrug-resistant organisms. *Enterococcus faecium* is an emergent nosocomial pathogen that is intrinsically resistant to aminoglycosides (kanamycin), tetracyclines, cephalosporins and quinolones, and that can acquire resistance to other antibiotics [31]. This very high occurrence of antibiotic resistance was shown also for dishwasher isolates. In Actinomycetales, *Micrococcus* spp. and the closely related genera are ubiquitous and are generally considered as harmless saprophytes that are relatively susceptible to most antibiotics. The majority of the *M. luteus* isolates from these dishwasher rubber seals were resistant to ciprofloxacin, which is contrary to the literature data (MIC, 0.8 μg/ml [73];), while the *Gordonia* isolates were susceptible to all of the antibiotics tested [4]. The *Brevibacterium casei* isolates should be susceptible to the majority of the antibiotics tested, except ciprofloxacin [65]; here *B. casei* was also resistant to cephalosporins and carbapenem antibiotics.

As representatives of Proteobacteria, most strains of *P. aeruginosa* are significantly more resistant to many antimicrobial agents than other closely related genera [22]. All of the dishwasher rubber seal isolates of *P. aeruginosa* were resistant to the tested carbapenems, cefotaxime and kanamycin, and some of them also to ciprofloxacin. Not surprisingly, only a few of other isolated pseudomonads, like *Acinetobacter* spp., where resistant to the carbapenems and/or cephalosporins tested [34]. Amongst the tested isolates of enterobacteria, the majority (*Klebsiella*, *Enterobacter*, *and Escherichia*) were susceptible to the tested antibiotics, except imipenem (*Enterobacter*) [5].

Amongst Xanthomonadales, *S. maltophilia* represents an emerging opportunistic pathogen, in particular due to its known resistance to many classes of antimicrobial agents [34]. All of these *S. maltophilia* dishwasher rubber seal isolates were resistant to all of the antibiotics tested. The Bacterioidetes *Chryseobacterium* spp. isolates are known to be intrinsically resistant to most β-lactams, including carbapenems, and to aminoglycosides, tetracyclines, fluoroquinolones and chloramphenicol [21], which was confirmed also in these dishwasher rubber seal isolates. Although the overall antibiotic resistance data of the dishwasher rubber seal bacterial isolates indicate that they do not represent a serious threat for the spread of antibiotic resistance into the household environment, dishwashers should nevertheless be considered as a potential source of infection with antibiotic resistant bacteria, in particular for immuno-compromised individuals.

The bacteria that colonise dishwashers can be released into the kitchens via aerosols and waste water, and by direct contact between contaminated surfaces and humans. Thus, dishwashers are possible sources of bacterial infections. Immuno-compromised patients with cystic fibrosis are an especially endangered group, particularly as they can often have chronic *P. aeruginosa* lung infections [12]. Although its deadliness is most apparent in patients with cystic fibrosis, *P. aeruginosa* is an opportunistic pathogen and therefore also a major problem in nosocomial infections in terms of burn and chronic wounds, chronic obstructive pulmonary disorder, surface growth on implanted biomaterials, and on hospital surfaces and in the water supply [7, 45, 49]. Amongst the 632 dishwasher rubber seal bacterial isolates, 12 were *P. aeruginosa*. Interiors of washing machines have been reported previously to harbour strains of *P. aeruginosa* [41], while this study also confirmed their presence in well-established biofilms on dishwasher rubber seals. Six out of 30 dishwashers were contaminated with *P. aeruginosa*, and these thus represented a major indoor environmental reservoir.

Another commonly encountered opportunistic pathogen *E. coli* was found in six out of 30 dishwashers examined. As *E. coli* strains are traditionally considered to be commensals of the microbiota in the intestinal tract of warm-blooded animals and humans, strains equipped with virulence factor genes can cause a wide spectrum of mild to severe extra-intestinal and intestinal infections [63]. Environmental *E. coli* strains are considered to arise primarily as a result of faecal contamination of soil, drinking water, recreational water, and groundwater [32]. Recent studies have suggested, however, that human opportunistic pathogenic *E. coli* strains can persist over longer periods of time as viable entities also in different hostile environments, especially when embedded in biofilms [32, 67]. To the best of our knowledge, this is the first description of *E. coli* isolates from biofilms that colonise dishwasher rubber seals. The primary source of these isolates might be both the household water supply system connected to the dishwasher and the contaminated vessels. Of interest also, the same sequence type of isolated *E. coli* strain was found in different dishwashers, which were even geographically located in different cities, thus indicating the strong selective pressure of this specific extreme environment. This has resulted in the enrichment of these not very virulent *E. coli* isolates, which have instead an emphasis on the ability to form adherent and persistent biofilms, and to take up sulphur and iron from the environment.

## Conclusion

We can conclude here that repeated mechanical, oxidative, water activity, and thermal stress inside dishwashers select for, and consequently enrich, biofilm-forming bacteria species, which in many instances are antibiotic resistant and virulent thermotolerant bacterial species. As these are the crucial factors that define most microbes in terms of their potential pathogenicity, as potential sources of human infections, domestic dishwashers cannot be ignored.

## Methods

### Sample collection

**Fig. 4:**
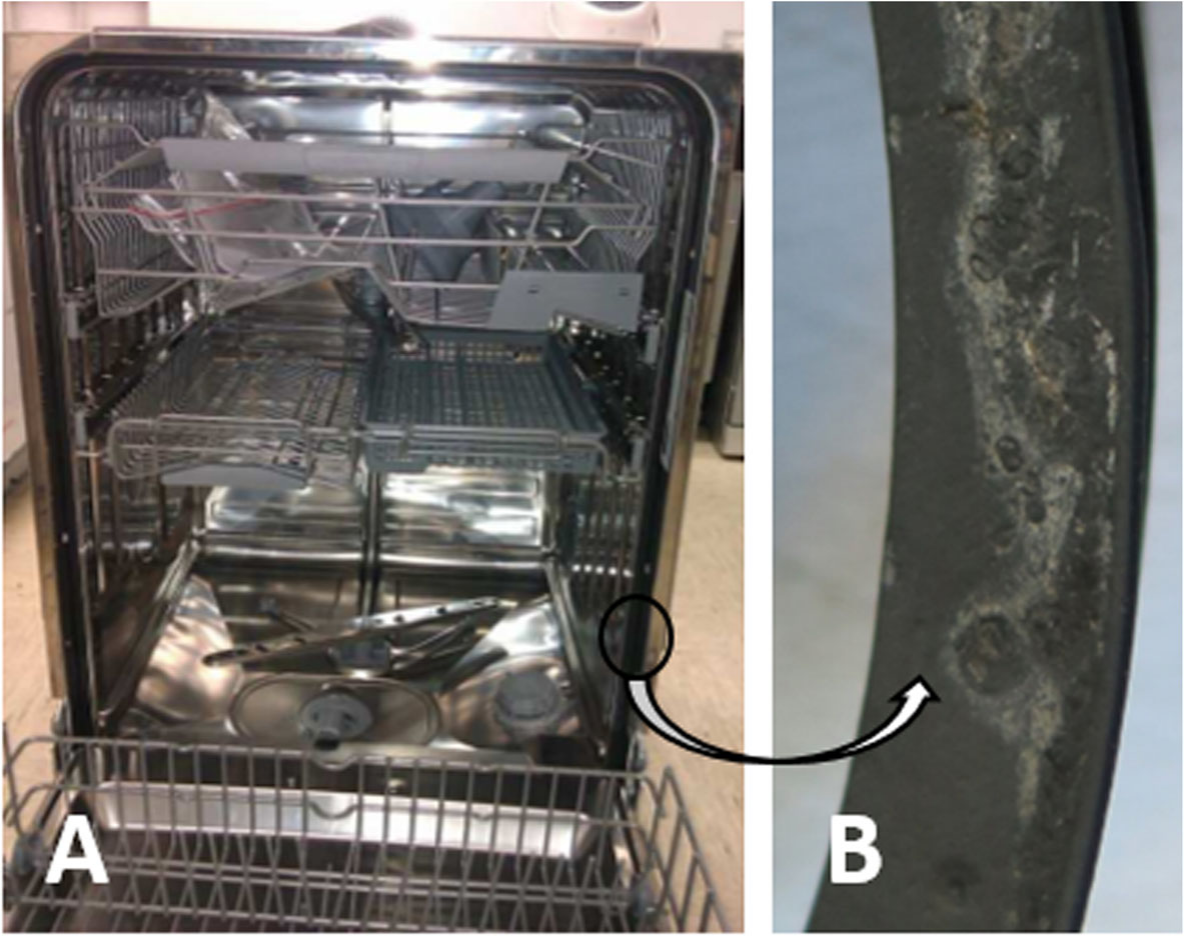
Dishwasher rubber seal. Sampling was performed in household dishawshers (**a**), on rubber seals where the outer edge of the dishwasher and the dishwasher door are in close contact (**b**)

For the sampling of the dishwasher seals, 30 dishwashers were randomly selected in kitchens inside private dwellings located in seven Slovenian cities (i.e., Ljubljana, Velenje, Žalec, Celje, Mislinja, Sežana, and Portorož). These dishwashers differed in age (1–8 years), brand (four different ones), frequency of use (once a week, to twice a day), and cleaning techniques (chemical, mechanical). Swab samples from their rubber seals (Fig. 4) were obtained by rubbing a cotton swab moistened with physiological saline over the seal surface at the end of the regular washing cycle. These swabs were immediately placed into sterile tubes, stored at 4 °C, and processed within 1 day. Further swab sampling was performed on washed vessels that had remained after washing in the dishwasher following an overnight wash cycle, such as glass lids of kitchenware, plastic kitchen boards, ceramic plates, and metal spoons. Additionally, 1.0 L tap water was taken from each of these 30 kitchens where these dishwashers were located. Sampling the biofilms formed on rubber seals of the 30 dishwashers was performed by scraping the seal surface with a sterile scalpel, and then placing the scraped material into sterile sampling tubes. The samples were stored at − 20 °C, and later combined following the DNA isolation.

### Isolation of bacterial isolates

For each swab, a separate agar plate was used. The swabs were streaked on nutrient agar, brain–heart infusion agar, Reasoner’s 2A agar, and M9 minimal medium [66]. These plates were incubated aerobically at 37 °C for 2 days (nutrient agar and brain–heart infusion agar) or for up to 7 days for minimal medium, and for 7 days at 37 °C for Reasoner’s 2A agar. For the isolation of anaerobes, swabs were streaked on brain–heart infusion agar plates and incubated anaerobically at 37 °C for 7 days. Colonies representing all of the morphotypes were re-streaked several times on the chosen medium to obtain pure cultures, which were deposited at the Ex Culture Collection, which is part of the Mycosmo Infrastructural Centre at the Department of Biology, Biotechnical Faculty, University of Ljubljana, Slovenia.

### Antibiotic resistance

All of the bacterial isolates were tested for resistance to a selection of antibiotics that was based on their importance in present curative treatments of stubborn bacterial infections. The seven chosen antibiotics were diluted in Lysogeny broth [LB] medium with agar, and used at the following final concentrations: 2 mg/L cefotaxime; 8 mg/L ceftazidime; 0.5 mg/L ertapenem; 2 mg/L imipenem; 0.25 mg/L ciprofloxacin; 15 mg/L tetracycline; and 50 mg/L kanamycin. The antibiotic solutions were sterilised by filtration (0.22 μm; Millipore) and added to LB agar medium cooled to 55 °C (in a water bath). The resistance against these antibiotics was checked with antibiotic susceptibility testing, with the bacterial isolates streaked to single colonies to LB agar plates with chosen antibiotics. Inoculated plates were incubated at 37 °C for up to 2 days (depending on the growth of the isolates). Additionally, all of these plates were incubated at 24 °C for another 2 days, and their growth was compared to the positive control (i.e., in LB agar plates without added antibiotics).

### Genomic DNA extraction and identification of bacterial isolates

Genomic DNA extraction was performed from overnight bacterial cultures grown on LB agar plates at 37 °C, using PrepMan Ultra Sample Preparation Reagent (Applied Biosystems), according to the manufacturer instructions.

The 16S rRNA genes were PCR amplified with oligonucleotide primers 27F (AGAGTTTGATCMTGGCTCAG [39];) and 1495r (CGGTTACCTTGTTACGACTT [2];). The PCR mixtures (35 μL) contained 1 μL isolated DNA, 0.45 U DreamTaq DNA polymerase (Thermo Fisher Scientific), 1× DreamTaq buffer with MgCl_2_ (Thermo Fisher Scientific), 0.1 mM dNTP (Thermo Fisher Scientific), and 0.1 μM of each primer. The reaction mixtures were first denatured at 94 °C for 5 min, and then subjected to 5 cycles of 94 °C for 30 s, 60 °C for 30 s, 72 °C for 1 min, 5 cycles of 94 °C for 30 s, 55 °C for 30 s, 72 °C for 1 min, and 30 cycles of 94 °C for 30 s, 50 °C for 30 s, and 72 °C for 1 min. Elongation in the last cycle lasted 7 min, followed by a final incubation at 4 °C. The PCR products were separated on 1% (w/v) agarose gels by electrophoresis in 1× TAE buffer, and subsequently purified and sequenced at Microsynth AG (Balgach, Switzerland) using the 27F sequencing primer.

The retrieved 16S rDNA sequences were identified on the basis of an approximately 800-bp-long amplicon, using the Ribosomal Database Project-II (RDP-II; http://rdp.cme.msu.edu) and National Centre for Biotechnology Information Basic Local Alignment Search Tool (NCBI BLAST) to search the GenBank non-redundant nucleotide database. Identification to the species level was defined as a 16S rDNA sequence similarity ≥99% with that of the prototype strain sequence in RDP-II; identification at the genus level was defined as a 16S rDNA sequence similarity ≥97% with that of the prototype strain sequence in RDP-II.

### Molecular and data analysis of biofilms and tap water

DNA from the biofilms from the scraping of the 30 sampled dishwasher rubber seals was isolated from 0.05 g to 0.1 g of biofilm biomass, using DNA isolation kit (Power Biofilm; MoBio, Carlsbad, CA, USA), according to the manufacturer instructions. Additionally, total DNA was isolated from the respective 30 tap water samples by filtering 1 L of water through 0.45-μm membrane filters (Merck, Millipore), and using DNA isolation kit (PowerWater; MoBio, Carlsbad, CA, USA), following the manufacturer instructions.

For the downstream sequencing, all 30 samples of these total DNA from biofilms were combined to a 5 ng/μL equimolar concentration and all of these 30 total DNA samples from water to a 3 ng/μL equimolar concentration. To target prokaryotic 16S rRNA genes for each of the pooled samples, PCR amplicon libraries were constructed using the 27F and 1495r bacterial primer sets [33]. Amplicon sequencing was carried out by Microsynth AG using a pyrosequencing platform (Roche 454). Initially, the sequences were quality trimmed with the threshold 25 and all reads shorter than 250 bp were removed. The reads were then processed bioinformatically with the QIIME software package [9]. The mean read length of the sequences was 535 bp, which covered the V1, V2 and V3 hyper-variable regions of 16S rDNA. Chimeric sequences were identified using the UCHIME algorithm [16] and discarded. Linker and reverse primers were trimmed. The maximum number of allowed homopolymers in a single bacterial sequence was set to six. The sequences were then clustered into operational taxonomic units (OTUs) by subsampling open reference clustering against the GreenGenes reference set, constructed at 97% similarity in the case of 16S rDNA analysis [38, 44]. The clustering was performed using the usearch61 algorithm [15] with 97% similarity preference as the standard definition of a bacterial species. Singletons were removed from further analysis. Alignments of the resulting 16S rDNA representative sequence sets were constructed using the ClustalX software [64]. Maximum likelihood methods implemented in PhyML 3.0 [26] were used to build phylogenetic trees to assign the taxonomy to new reference OTUs where possible. When the reference collections did not yield any results, taxonomy assignment was attempted using UNITE+INSD (International Nucleotide Sequence Databases: National Centre for Biotechnology Information; *European Molecular Biology Laboratory*; *DNA Data Bank of Japan*).

## Supplementary information

**Additional file 1: Table S1.** Characteristics of the individual dishwashers sampled. **Table S2.** Bacterial phyla detected in the water and biofilm samples. **Table S3.** Antibiogram results, showing all of the isolated bacteria from the rubber seals of the 30 residential dishwashers in terms of their antibiotic resistance against the chosen antibiotics.

## Data Availability

All data generated or analysed during this study are included in this published article (and its supplementary information files).

## Abbreviations

CFZ: ceftazidime
CIP: ciprofloxacin
CTX: cefotaxime
ETP: ertapenem
IMP: imipenem
KN: kanamycin
LB: Lysogeny broth medium with agar
MIC: Minimum inhibitory concentration
OTUs: operational taxonomic units
RDP-II: the Ribosomal Database Project-II
TC: tetracycline
