# Supplementary material for: The dishwasher rubber seal acts as a reservoir of bacteria in the home environment
Source: BMC Microbiol. 2019 Dec 19;19:300. doi: 10.1186/s12866-019-1674-5 (PMC6924085; doi:10.1186/s12866-019-1674-5)
Supplement: Supplementary file 1 — Additional file 1: Table S1. Characteristics of the individual dishwashers sampled. Table S2. Bacterial phyla detected in the water and biofilm samples. Table S3. Antibiogram results, showing all of the isolated bacteria from the rubber seals of the 30 residential dishwashers in terms of their antibiotic resistance against the chosen antibiotics. [file 12866_2019_1674_MOESM1_ESM.docx]

**Additional file**

**Table S1.** Characteristics of the individual dishwashers sampled.

| **Dishwasher** | **Time of use (years)** | **Frequency of use (times per week)** | **Cleaning** | **Washing temperature (°C)** | **Water hardness *** | **Water CaCO_3_ concentration (mmol/L)** | **Different species isolated from rubber seals** |
| --- | --- | --- | --- | --- | --- | --- | --- |
| 1 | 2.5 | 7 | Chemical | 60 | Slightly hard | 0.963 | 8 |
| 2 | 2.5 | 7 | None | 60 | Slightly hard | 1.163 | 12 |
| 3 | 1.5 | 7 | Chemical | 60 | Hard | 1.817 | 5 |
| 4 | 7 | 3 | Chemical | 60 | Moderately hard | 1.320 | 11 |
| 5 | 3 | 14 | Chemical | 60 | Moderately hard | 1.664 | 8 |
| 6 | 2 | 14 | Chemical | 60 | Moderately hard | 1.612 | 22 |
| 7 | ND | ND | ND | ND | ND | ND | 4 |
| 8 | 8 | 7 | None | 60 | Moderately hard | 1.440 | 5 |
| 9 | 6 | 7 | None | 60 | Slightly hard | 0.871 | 7 |
| 10 | 5 | 3 | None | 60 | Moderately hard | 1.497 | 17 |
| 11 | 8 | 7 | None | 60 | Moderately hard | 1.218 | 6 |
| 12 | 5 | 3 | None | 60 | Moderately hard | 1.390 | 4 |
| 13 | 1 | 2 | None | 65 | Moderately hard | 1.293 | 9 |
| 14 | 3 | 14 | Chemical | 70 | Hard | 1.984 | 2 |
| 15 | 7 | 7 | None | 60 | Slightly hard | 1.113 | 8 |
| 19 | 0.5 | 3 | Mechanical | 65 | Slightly hard | 1.095 | 7 |
| 20 | 1 | 3 | None | 50 | Slightly hard | 0.963 | 14 |
| 21 | 2 | 2 | Chemical | 65 | Slightly hard | 1.003 | 5 |
| 22 | 8 | 7 | None | 65 | Moderately soft | 0.394 | 11 |
| 23 | 1 | 7 | Chemical | 65 | Moderately hard | 1.263 | 8 |
| 24 | 0.5 | 14 | None | 50 | Moderately hard | 1.317 | 12 |
| 25 | 1 | 14 | Chemical | 65 | Moderately hard | 1.495 | 11 |
| 27 | 1.5 | 7 | None | 65 | Moderately hard | 1.325 | 7 |
| 28 | 4 | 4 | None | 65 | Moderately hard | 1.774 | 5 |
| 29 | 0.5 | 7 | Mechanical | 65 | Hard | 2.054 | 15 |
| 30 | 4 | 3 | None | 65 | Hard | 1.991 | 11 |
| 31 | 3 | 1 | None | 60 | Slightly hard | 1.203 | 9 |
| 32 | ND | ND | ND | ND | Moderately soft | 0.512 | 4 |
| 33 | 8 | 7 | Chemical | 50 | Moderately soft | 0.521 | 5 |
| 34 | 5 | 7 | None | 60 | Hard | 1.856 | 6 |
| 35 | 1 | 1 | None | 65 | Slightly hard | 0.953 | 1 |

ND, no data

*Water hardness ratings: Soft, ≤0.169 mmol/L CaCO_3_; moderately soft, 0.170-0.597 mmol/L CaCO_3_; slightly hard, 0.598-1.496 mmol/L CaCO_3_; moderately hard, 1.497-1.792 mmol/L CaCO_3_; hard, 1.793-2.213 mmol/L CaCO_3_; very hard, ≥2,214 mmol/L CaCO_3_

**Table S2.** Bacterial phyla detected in the water and biofilm samples.

| **Phylum** | **Class** | **Order** | **Family** | **Genus** | **Species** | **OTU** | |
| --- | --- | --- | --- | --- | --- | --- | --- |
|  |  |  |  |  |  | **Biofilm** | **Water** |
| Actinobacteria | Actinobacteria | Actinomycetales | *Dermabacteraceae* | *Brachybacterium* | sp. | 10 | 1 |
|  |  |  | *Dietziaceae* | *Dietzia* | sp. | 66 | 0 |
|  |  |  | *Gordoniaceae* | *Gordonia* | sp. | 2142 | 7 |
|  |  |  | *Intrasporangiaceae* | *Ornithinicoccus* | sp. | 90 | 0 |
|  |  |  | *Microbacteriaceae* | *Kocuria* | sp. | 18 | 0 |
|  |  |  |  | *Microbacterium* | *lacticum* | 20 | 0 |
|  |  |  |  | *Microbacterium* | sp. | 153 | 0 |
|  |  |  |  | *Pseudoclavibacter* | sp. | 9 | 0 |
|  |  |  | *Micrococcaceae* | *Arthrobacter* | *nicotinovorans* | 9 | 0 |
|  |  |  |  | *Arthrobacter* | sp. | 19 | 0 |
|  |  |  |  | *Micrococcus* | luteus | 73 | 0 |
|  |  |  |  | *Micrococcus* | sp. | 23 | 0 |
|  |  |  |  | *Nesterenkonia* | *lacusekhoensis* | 58 | 0 |
|  |  |  |  | *Nesterenkonia* | sp. | 199 | 0 |
|  |  |  | *Micrococcaceae* |  |  | 7 | 0 |
|  |  |  | *Mycobacteriaceae* | *Mycobacterium* | sp. | 2 | 25 |
|  |  |  | *Nocardioidaceae* | *Aeromicrobium* | sp. | 40 | 0 |
|  |  |  | *Propionibacteriaceae* | *Propionibacterium* | sp. | 47 | 0 |
|  |  |  |  | *Propioniciclava* | sp. | 19 | 0 |
|  |  |  |  | *Tessaracoccus* | sp. | 9 | 0 |
|  |  |  | *Propionibacteriaceae* |  |  | 118 | 0 |
|  |  | Actinomycetales |  |  |  | 57 | 0 |
|  |  | Solirubrobacterales |  |  |  | 41 | 0 |
|  | Actinobacteria |  |  |  |  | 7 | 0 |
| Chloroflexi | Thermomicrobia |  |  |  |  | 21 | 0 |
| Cyanobacteria |  |  |  |  |  | 3 | 12 |
| Deinococcus- | Deinococci | Thermales | *Thermaceae* | *Meiothermus* | sp. | 46 | 0 |
| Thermus |  |  |  | *Thermus* | sp. | 0 | 15 |
| Firmicutes | Bacilli | Bacillales | *Bacillaceae* | *Bacillus* | sp. | 74 | 0 |
|  |  |  | *Bacillales-Incertae Sedis XII* | *Exiguobacterium* | sp. | 128 | 0 |
|  |  |  |  | *Exiguobacterium* | *mexicanum* | 40 | 0 |
|  |  | Bacillales |  |  |  | 11 | 0 |
| Proteobacteria | Alphaproteobacteria | Caulobacterales | *Caulobacteraceae* | *Brevundimonas* | sp. | 9 | 6 |
|  |  |  |  | *Phenylobacterium* | sp. | 0 | 18 |
|  |  | Rhizobiales | *Bradyrhizobiaceae* | *Bosea* | sp. | 2 | 7 |
|  |  |  |  | *Bradyrhizobium* | sp. | 1 | 7 |
|  |  |  | *Brucellaceae* | *Ochrobactrum* | sp. | 8 | 0 |
|  |  |  | *Methylobacteriaceae* | *Methylobacterium* | sp. | 0 | 30 |
|  |  |  | *Xanthobacteraceae* | *Starkeya* | sp. | 7 | 0 |
|  |  |  |  |  |  | 5 | 12 |
|  |  | Rhodobacterales | *Rhodobacteraceae* | *Paracoccus* | sp. | 18 | 0 |
|  |  |  |  | *Rhodobacter* | sp. | 10 | 0 |
|  |  | Rhodobacterales |  |  |  | 17 | 0 |
|  |  | Sphingomonadales | *Sphingomonadaceae* | *Blastomonas* | sp. | 0 | 66 |
|  |  |  |  | *Novosphingobium* | sp. | 1 | 27 |
|  |  |  | *Sphingomonadaceae* |  | sp. | 0 | 25 |
|  |  |  | *Sphingomonadaceae* | *Sphingomonas* | sp. | 6 | 92 |
|  |  |  |  | *Sphingomonas* | sp. GOBB3-C201 | 0 | 39 |
|  |  |  |  | *Sphingopyxis* | *macrogoltabida* | 0 | 7 |
|  |  |  |  | *Sphingopyxis* | sp. | 15 | 16 |
|  | Betaproteobacteria | Burkholderiales | *Burkholderiales Genera incertae sedis* | *Piscinibacter* | sp. | 1 | 22 |
|  |  |  |  | *Tepidimonas* | sp. | 5 | 853 |
|  |  | Burkholderiales |  |  |  | 4 | 30 |
|  |  | Hydrogenophilales | *Hydrogenophilaceae* | *Petrobacter* | sp. | 65 | 0 |
|  |  | Rhodocyclales | *Rhodocyclaceae* | *Ferribacterium* | sp. | 0 | 7 |
|  |  |  |  | *Methyloversatilis* | sp. | 0 | 9 |
|  |  |  |  | *Sulfuritalea* | sp. | 0 | 8 |
|  | Gammaproteobacteria | Enterobacteriales | *Enterobacteriaceae* | *Enterobacter* | sp. | 11 | 0 |
|  |  |  |  | *Escherichia/Shigella* | sp. | 44 | 0 |
|  |  | Pseudomonadales | *Moraxellaceae* | *Acinetobacter* | *baumanii* | 17 | 0 |
|  |  |  |  | *Acinetobacter* | sp. | 199 | 0 |
|  |  |  |  | *Perlucidibaca* | sp. | 0 | 78 |
|  |  |  | *Pseudomonadaceae* | *Pseudomonas* | sp. | 44 | 0 |
|  |  | Pseudomonadales |  |  |  | 8 | 0 |
|  |  | Thiotrichales |  |  |  | 0 | 11 |
|  |  | Xanthomonadales | *Sinobacteraceae* | *Nevskia* | *ramosa* | 0 | 7 |
|  |  |  |  | *Nevskia* | sp. | 0 | 34 |
|  |  |  | *Xanthomonadaceae* | *Dokdonella* | sp. | 14 | 1 |
|  |  |  |  | *Pseudoxanthomonas* | sp. | 35 | 1 |
|  |  |  |  | *Stenotrophomonas* | *maltophilia* | 13 | 0 |
|  |  |  |  | *Stenotrophomonas* | sp. | 64 | 0 |
|  |  |  |  | *Xylella* | sp. | 16 | 0 |
|  | Gammaproteobacteria |  |  |  |  | 2 | 7 |
| Candidate division BRC11 |  |  |  |  |  | 36 | 0 |
| Candidatus Saccharibacteria |  |  |  |  |  | 257 | 0 |
| Unassigned |  |  |  |  |  | 135 | 23 |
| Unidentified |  |  |  |  |  | 10 | 0 |
| **TOTAL** |  |  |  |  |  | **4638** | **1503** |

**Table S3.** Antibiogram results, showing all of the isolated bacteria from the rubber seals of the 30 residential dishwashers in terms of their antibiotic resistance against the chosen antibiotics. Tested concentrations were: 2 mg/L cefotaxime (CTX); 8 mg/L ceftazidime (CFZ); 0.5 mg/L ertapenem (ETP); 2 mg/L imipenem (IMP); 0.25 mg/L ciprofloxacin (CIP); 15 mg/L tetracycline (TC); and 50 mg/L kanamycin (KN). LB medium, no-antibiotic control. +, growth; -, inhibition.

| **EXB No.** | ***Bacterium*** | **Phylum, class, order, family** | **Dishwasher** | **CTX** | **CFZ** | **ETP** | **IMP** | **CIP** | **TC** | **KN** | **LB medium** |
| --- | --- | --- | --- | --- | --- | --- | --- | --- | --- | --- | --- |
| **L-406** | *Achromobacter insolitus* | Proteobacteria, Betaproteobacteria, Burkholderiales, Alcaligenaceae | 10 | **+** |  |  |  |  | **+** | **-** | **+** |
| **L-694** | *Acinetobacter calcoaceticus* | Proteobacteria, Gammaproteobacteria, Pseudomonadales, Moraxellaceae | 6 | **+** | **-** | **+** | **-** | **-** | **-** | **-** | **+** |
| **L-693** | *Acinetobacter calcoaceticus* | Proteobacteria, Gammaproteobacteria, Pseudomonadales, Moraxellaceae | 6 | **+** | **-** | **+** | **-** | **-** | **-** | **-** | **+** |
| **L-411** | *Acinetobacter calcoaceticus* | Proteobacteria, Gammaproteobacteria, Pseudomonadales, Moraxellaceae | 6 | **+** | **-** | **+** | **-** | **-** | **-** | **-** | **+** |
| **L-527** | *Acinetobacter calcoaceticus* | Proteobacteria, Gammaproteobacteria, Pseudomonadales, Moraxellaceae | 2 | **+** | **+** | **+** | **-** | **+** | **-** | **-** | **+** |
| **L-963** | *Acinetobacter calcoaceticus* | Proteobacteria, Gammaproteobacteria, Pseudomonadales, Moraxellaceae | 13 | **+** | **+** | **+** | **+** | **+** | **-** | **+** | **+** |
| **L-381** | *Acinetobacter junii* | Proteobacteria, Gammaproteobacteria, Pseudomonadales, Moraxellaceae | 4 | **+** |  |  |  |  | **-** | **-** | **+** |
| **L-386** | *Acinetobacter junii* | Proteobacteria, Gammaproteobacteria, Pseudomonadales, Moraxellaceae | 4 | **-** |  |  |  |  | **-** | **-** | **+** |
| **L-528** | *Acinetobacter* sp. | Proteobacteria, Gammaproteobacteria, Pseudomonadales, Moraxellaceae | 19 | **+** | **+** | **+** | **-** | **-** | **-** | **-** | **+** |
| **L-724** | *Acinetobacter sp.* | Proteobacteria, Gammaproteobacteria, Pseudomonadales, Moraxellaceae | 19 | **+** | **-** | **-** | **-** | **-** | **-** | **-** | **+** |
| **L-838** | *Acinetobacter* sp. | Proteobacteria, Gammaproteobacteria, Pseudomonadales, Moraxellaceae | 19 | **+** | **-** | **-** | **-** | **-** | **-** | **-** | **+** |
| **L-698** | *Acinetobacter* sp. | Proteobacteria, Gammaproteobacteria, Pseudomonadales, Moraxellaceae | 20 | **+** | **+** | **+** | **-** | **-** | **-** | **-** | **+** |
| **L-958** | *Acinetobacter* sp. | Proteobacteria, Gammaproteobacteria, Pseudomonadales, Moraxellaceae | 20 | **+** | **+** | **+** | **-** | **-** | **-** | **-** | **+** |
| **L-391** | *Acinetobacter* sp. | Proteobacteria, Gammaproteobacteria, Pseudomonadales, Moraxellaceae | 6 | **+** |  |  | **-** | **-** | **-** | **-** | **+** |
| **L-397** | *Acinetobacter* sp. | Proteobacteria, Gammaproteobacteria, Pseudomonadales, Moraxellaceae | 6 | **+** |  |  | **-** | **-** | **-** | **-** | **+** |
| **L-935** | *Acinetobacter ursingii* | Proteobacteria, Gammaproteobacteria, Pseudomonadales, Moraxellaceae | 20 | **+** | **+** | **+** | **-** | **-** | **-** | **-** | **+** |
| **L-529** | *Aerococcus* sp. | Firmicutes, Bacilli, Lactobacillales, Aerococcaceae | 22 | **+** | **+** | **-** | **-** | **+** | **-** | **+** | **+** |
| **L-765** | *Aerococcus* sp. | Firmicutes, Bacilli, Lactobacillales, Aerococcaceae | 15 | **-** | **-** | **-** | **-** | **+** | **-** | **-** | **+** |
| **L-763** | *Aerococcus viridans* | Firmicutes, Bacilli, Lactobacillales, Aerococcaceae | 15 | **-** | **-** | **-** | **-** | **+** | **-** | **-** | **+** |
| **L-764** | *Aerococcus viridans* | Firmicutes, Bacilli, Lactobacillales, Aerococcaceae | 15 | **-** | **+** | **-** | **-** | **+** | **-** | **-** | **+** |
| **L-766** | *Aerococcus viridans* | Firmicutes, Bacilli, Lactobacillales, Aerococcaceae | 15 | **-** | **-** | **-** | **-** | **+** | **-** | **-** | **+** |
| **L-925** | *Aerococcus viridans* | Firmicutes, Bacilli, Lactobacillales, Aerococcaceae | 15 | **-** | **-** | **-** | **-** | **+** | **-** | **-** | **+** |
| **L-1063** | *Aerococcus viridans* | Firmicutes, Bacilli, Lactobacillales, Aerococcaceae | 22 | **-** | **-** | **-** | **-** | **+** | **-** | **-** | **+** |
| **L-954** | *Aeromicrobium* sp. | Actinobacteria, Actinobacteria, Actinomycetales, Nocardioidaceae | 6 | **-** | **-** | **-** | **-** | **+** | **-** | **+** | **+** |
| **L-870** | *Rhizobium radiobacter* | Proteobacteria, Alphaproteobacteria, Rhizobiales, Rhizobiaceae | 24 | **+** | **+** | **-** | **-** | **-** | **-** | **+** | **+** |
| **L-949** | *Rhizobium radiobacter* | Proteobacteria, Alphaproteobacteria, Rhizobiales, Rhizobiaceae | 24 | **+** | **+** | **-** | **-** | **-** | **-** | **+** | **+** |
| **L-1011** | *Rhizobium radiobacter* | Proteobacteria, Alphaproteobacteria, Rhizobiales, Rhizobiaceae | 24 | **+** | **+** | **-** | **-** | **-** | **-** | **+** | **+** |
| **L-1012** | *Rhizobium radiobacter* | Proteobacteria, Alphaproteobacteria, Rhizobiales, Rhizobiaceae | 24 | **+** | **+** | **-** | **-** | **-** | **-** | **+** | **+** |
| **L-1020** | *Rhizobium radiobacter* | Proteobacteria, Alphaproteobacteria, Rhizobiales, Rhizobiaceae | 29 | **+** | **+** | **-** | **-** | **-** | **-** | **+** | **+** |
| **L-707** | *Bacillus amyloliquefaciens* | Firmicutes, Bacilli, Bacillales, Bacillaceae | 14 | **-** | **-** | **-** | **-** | **-** | **-** | **-** | **+** |
| **L-708** | *Bacillus amyloliquefaciens* | Firmicutes, Bacilli, Bacillales, Bacillaceae | 14 | **-** | **-** | **-** | **-** | **-** | **-** | **-** | **+** |
| **L-688** | *Bacillus amyloliquefaciens* | Firmicutes, Bacilli, Bacillales, Bacillaceae | 25 | **-** | **-** | **-** | **-** | **-** | **-** | **-** | **+** |
| **L-908** | *Bacillus amyloliquefaciens* | Firmicutes, Bacilli, Bacillales, Bacillaceae | 30 | **+** | **+** | **-** | **-** | **-** | **-** | **-** | **+** |
| **L-909** | *Bacillus amyloliquefaciens* | Firmicutes, Bacilli, Bacillales, Bacillaceae | 30 | **+** | **+** | **-** | **-** | **-** | **-** | **-** | **+** |
| **L-914** | *Bacillus amyloliquefaciens* | Firmicutes, Bacilli, Bacillales, Bacillaceae | 30 | **+** | **+** | **-** | **-** | **-** | **-** | **-** | **+** |
| **L-915** | *Bacillus amyloliquefaciens* | Firmicutes, Bacilli, Bacillales, Bacillaceae | 30 | **+** | **+** | **-** | **-** | **-** | **-** | **-** | **+** |
| **L-916** | *Bacillus amyloliquefaciens* | Firmicutes, Bacilli, Bacillales, Bacillaceae | 30 | **+** | **+** | **-** | **-** | **-** | **-** | **-** | **+** |
| **L-452** | *Bacillus cereus* group | Firmicutes, Bacilli, Bacillales, Bacillaceae | 1 | **+** | **+** | **-** | **-** | **-** | **-** | **-** | **+** |
| **L-466** | *Bacillus cereus* group | Firmicutes, Bacilli, Bacillales, Bacillaceae | 1 | **+** | **+** | **-** | **-** | **-** | **-** | **-** | **+** |
| **L-441** | *Bacillus cereus* group | Firmicutes, Bacilli, Bacillales, Bacillaceae | 10 | **+** | **+** | **-** | **-** | **-** | **-** | **-** | **+** |
| **L-402** | *Bacillus cereus* group | Firmicutes, Bacilli, Bacillales, Bacillaceae | 10 | **+** | **+** | **-** | **-** | **-** | **-** | **-** | **+** |
| **L-841** | *Bacillus cereus* group | Firmicutes, Bacilli, Bacillales, Bacillaceae | 10 | **+** | **+** | **-** | **-** | **-** | **-** | **-** | **+** |
| **L-577** | *Bacillus cereus* group | Firmicutes, Bacilli, Bacillales, Bacillaceae | 10 | **+** | **+** | **-** | **-** | **-** | **-** | **-** | **+** |
| **L-703** | *Bacillus cereus* group | Firmicutes, Bacilli, Bacillales, Bacillaceae | 10 | **+** | **+** | **-** | **-** | **-** | **-** | **-** | **+** |
| **L-725** | *Bacillus cereus* group | Firmicutes, Bacilli, Bacillales, Bacillaceae | 10 | **+** | **+** | **-** | **-** | **-** | **-** | **-** | **+** |
| **L-432** | *Bacillus cereus* group | Firmicutes, Bacilli, Bacillales, Bacillaceae | 10 | **+** | **+** | **-** | **-** | **-** | **-** | **-** | **+** |
| **L-697** | *Bacillus cereus* group | Firmicutes, Bacilli, Bacillales, Bacillaceae | 10 | **+** | **+** | **-** | **-** | **-** | **-** | **-** | **+** |
| **L-771** | *Bacillus cereus* group | Firmicutes, Bacilli, Bacillales, Bacillaceae | 11 | **+** | **+** | **-** | **-** | **-** | **-** | **-** | **+** |
| **L-767** | *Bacillus cereus* group | Firmicutes, Bacilli, Bacillales, Bacillaceae | 11 | **+** | **+** | **-** | **-** | **-** | **-** | **-** | **+** |
| **L-760** | *Bacillus cereus* group | Firmicutes, Bacilli, Bacillales, Bacillaceae | 15 | **+** | **+** | **-** | **-** | **-** | **-** | **-** | **+** |
| **L-442** | *Bacillus cereus* group | Firmicutes, Bacilli, Bacillales, Bacillaceae | 19 | **+** | **+** | **-** | **-** | **-** | **-** | **-** | **+** |
| **L-443** | *Bacillus cereus* group | Firmicutes, Bacilli, Bacillales, Bacillaceae | 19 | **+** | **+** | **-** | **-** | **-** | **-** | **-** | **+** |
| **L-444** | *Bacillus cereus* group | Firmicutes, Bacilli, Bacillales, Bacillaceae | 19 | **+** | **+** | **-** | **-** | **-** | **-** | **-** | **+** |
| **L-823** | *Bacillus cereus* group | Firmicutes, Bacilli, Bacillales, Bacillaceae | 19 | **+** | **+** | **-** | **-** | **-** | **-** | **-** | **+** |
| **L-825** | *Bacillus cereus* group | Firmicutes, Bacilli, Bacillales, Bacillaceae | 19 | **+** | **+** | **-** | **-** | **-** | **-** | **-** | **+** |
| **L-837** | *Bacillus cereus* group | Firmicutes, Bacilli, Bacillales, Bacillaceae | 19 | **+** | **+** | **-** | **-** | **-** | **-** | **-** | **+** |
| **L-468** | *Bacillus cereus* group | Firmicutes, Bacilli, Bacillales, Bacillaceae | 19 | **+** | **+** | **-** | **-** | **-** | **-** | **-** | **+** |
| **L-640** | *Bacillus cereus* group | Firmicutes, Bacilli, Bacillales, Bacillaceae | 20 | **+** | **+** | **-** | **-** | **-** | **+** | **-** | **+** |
| **L-450** | *Bacillus cereus* group | Firmicutes, Bacilli, Bacillales, Bacillaceae | 20 | **+** | **+** | **-** | **-** | **-** | **-** | **-** | **+** |
| **L-675** | *Bacillus cereus* group | Firmicutes, Bacilli, Bacillales, Bacillaceae | 20 | **+** | **+** | **-** | **-** | **-** | **-** | **-** | **+** |
| **L-1027** | *Bacillus cereus* group | Firmicutes, Bacilli, Bacillales, Bacillaceae | 20 | **+** | **+** | **-** | **-** | **-** | **+** | **-** | **+** |
| **L-1042** | *Bacillus cereus* group | Firmicutes, Bacilli, Bacillales, Bacillaceae | 20 | **+** | **+** | **-** | **-** | **-** | **-** | **-** | **+** |
| **L-467** | *Bacillus cereus* group | Firmicutes, Bacilli, Bacillales, Bacillaceae | 20 | **+** | **+** | **-** | **-** | **-** | **-** | **-** | **+** |
| **L-445** | *Bacillus cereus* group | Firmicutes, Bacilli, Bacillales, Bacillaceae | 21 | **+** | **+** | **-** | **-** | **-** | **-** | **-** | **+** |
| **L-449** | *Bacillus cereus* group | Firmicutes, Bacilli, Bacillales, Bacillaceae | 21 | **+** | **+** | **-** | **-** | **-** | **-** | **-** | **+** |
| **L-454** | *Bacillus cereus* group | Firmicutes, Bacilli, Bacillales, Bacillaceae | 21 | **+** | **+** | **-** | **-** | **-** | **-** | **-** | **+** |
| **L-457** | *Bacillus cereus* group | Firmicutes, Bacilli, Bacillales, Bacillaceae | 21 | **+** | **+** | **-** | **-** | **-** | **-** | **-** | **+** |
| **L-464** | *Bacillus cereus* group | Firmicutes, Bacilli, Bacillales, Bacillaceae | 22 | **+** | **+** | **-** | **-** | **-** | **-** | **-** | **+** |
| **L-525** | *Bacillus cereus* group | Firmicutes, Bacilli, Bacillales, Bacillaceae | 24 | **+** | **+** | **-** | **-** | **-** | **-** | **-** | **+** |
| **L-847** | *Bacillus cereus* group | Firmicutes, Bacilli, Bacillales, Bacillaceae | 27 | **+** | **+** | **-** | **-** | **-** | **-** | **-** | **+** |
| **L-455** | *Bacillus cereus* group | Firmicutes, Bacilli, Bacillales, Bacillaceae | 27 | **+** | **+** | **-** | **-** | **-** | **-** | **-** | **+** |
| **L-456** | *Bacillus cereus* group | Firmicutes, Bacilli, Bacillales, Bacillaceae | 27 | **+** | **+** | **-** | **-** | **-** | **-** | **-** | **+** |
| **L-459** | *Bacillus cereus* group | Firmicutes, Bacilli, Bacillales, Bacillaceae | 27 | **+** | **+** | **-** | **-** | **-** | **-** | **-** | **+** |
| **L-470** | *Bacillus cereus* group | Firmicutes, Bacilli, Bacillales, Bacillaceae | 27 | **+** | **+** | **-** | **-** | **-** | **-** | **-** | **+** |
| **L-471** | *Bacillus cereus* group | Firmicutes, Bacilli, Bacillales, Bacillaceae | 27 | **+** | **+** | **-** | **-** | **-** | **-** | **-** | **+** |
| **L-792** | *Bacillus cereus* group | Firmicutes, Bacilli, Bacillales, Bacillaceae | 28 | **+** | **+** | **-** | **-** | **-** | **-** | **-** | **+** |
| **L-461** | *Bacillus cereus* group | Firmicutes, Bacilli, Bacillales, Bacillaceae | 29 | **+** | **+** | **-** | **-** | **-** | **-** | **-** | **+** |
| **L-462** | *Bacillus cereus* group | Firmicutes, Bacilli, Bacillales, Bacillaceae | 29 | **+** | **+** | **-** | **-** | **-** | **-** | **-** | **+** |
| **L-447** | *Bacillus cereus* group | Firmicutes, Bacilli, Bacillales, Bacillaceae | 3 | **+** | **+** | **-** | **-** | **+** | **-** | **-** | **+** |
| **L-448** | *Bacillus cereus* group | Firmicutes, Bacilli, Bacillales, Bacillaceae | 3 | **+** | **+** | **-** | **-** | **-** | **-** | **-** | **+** |
| **L-472** | *Bacillus cereus* group | Firmicutes, Bacilli, Bacillales, Bacillaceae | 3 | **+** | **+** | **-** | **-** | **-** | **-** | **-** | **+** |
| **L-854** | *Bacillus cereus* group | Firmicutes, Bacilli, Bacillales, Bacillaceae | 30 | **+** | **+** | **-** | **-** | **-** | **-** | **-** | **+** |
| **L-458** | *Bacillus cereus* group | Firmicutes, Bacilli, Bacillales, Bacillaceae | 30 | **+** | **+** | **-** | **-** | **-** | **-** | **-** | **+** |
| **L-460** | *Bacillus cereus* group | Firmicutes, Bacilli, Bacillales, Bacillaceae | 30 | **+** | **+** | **-** | **-** | **-** | **-** | **-** | **+** |
| **L-913** | *Bacillus cereus* group | Firmicutes, Bacilli, Bacillales, Bacillaceae | 31 | **+** | **+** | **-** | **-** | **-** | **-** | **-** | **+** |
| **L-819** | *Bacillus cereus* group | Firmicutes, Bacilli, Bacillales, Bacillaceae | 32 | **+** | **+** | **-** | **-** | **-** | **-** | **-** | **+** |
| **L-660** | *Bacillus cereus* group | Firmicutes, Bacilli, Bacillales, Bacillaceae | 32 | **+** | **+** | **-** | **-** | **-** | **-** | **-** | **+** |
| **L-661** | *Bacillus cereus* group | Firmicutes, Bacilli, Bacillales, Bacillaceae | 32 | **+** | **+** | **-** | **-** | **-** | **-** | **-** | **+** |
| **L-818** | *Bacillus cereus* group | Firmicutes, Bacilli, Bacillales, Bacillaceae | 32 | **+** | **+** | **-** | **-** | **-** | **-** | **-** | **+** |
| **L-806** | *Bacillus cereus* group | Firmicutes, Bacilli, Bacillales, Bacillaceae | 33 | **+** | **+** | **-** | **-** | **-** | **-** | **-** | **+** |
| **L-807** | *Bacillus cereus* group | Firmicutes, Bacilli, Bacillales, Bacillaceae | 33 | **+** | **+** | **-** | **-** | **-** | **-** | **-** | **+** |
| **L-642** | *Bacillus cereus* group | Firmicutes, Bacilli, Bacillales, Bacillaceae | 33 | **+** | **+** | **-** | **-** | **-** | **-** | **-** | **+** |
| **L-643** | *Bacillus cereus* group | Firmicutes, Bacilli, Bacillales, Bacillaceae | 33 | **+** | **+** | **-** | **-** | **-** | **-** | **-** | **+** |
| **L-918** | *Bacillus cereus* group | Firmicutes, Bacilli, Bacillales, Bacillaceae | 34 | **+** | **+** | **-** | **-** | **-** | **-** | **-** | **+** |
| **L-649** | *Bacillus cereus* group | Firmicutes, Bacilli, Bacillales, Bacillaceae | 34 | **+** | **+** | **-** | **-** | **-** | **-** | **-** | **+** |
| **L-379** | *Bacillus cereus* group | Firmicutes, Bacilli, Bacillales, Bacillaceae | 4 | **+** | **+** | **-** | **-** | **-** | **-** | **-** | **+** |
| **L-383** | *Bacillus cereus group* | Firmicutes, Bacilli, Bacillales, Bacillaceae | 4 | **+** | **+** | **-** | **-** | **-** | **-** | **-** | **+** |
| **L-407** | *Bacillus cereus* group | Firmicutes, Bacilli, Bacillales, Bacillaceae | 4 | **+** | **+** | **-** | **-** | **-** | **-** | **-** | **+** |
| **L-446** | *Bacillus cereus* group | Firmicutes, Bacilli, Bacillales, Bacillaceae | 4 | **+** | **+** | **-** | **-** | **-** | **-** | **-** | **+** |
| **L-453** | *Bacillus cereus* group | Firmicutes, Bacilli, Bacillales, Bacillaceae | 4 | **+** | **+** | **-** | **-** | **-** | **-** | **-** | **+** |
| **L-451** | *Bacillus cereus* group | Firmicutes, Bacilli, Bacillales, Bacillaceae | 5 | **+** | **+** | **-** | **-** | **-** | **-** | **-** | **+** |
| **L-576** | *Bacillus cereus* group | Firmicutes, Bacilli, Bacillales, Bacillaceae | 5 | **+** | **+** | **-** | **-** | **-** | **-** | **-** | **+** |
| **L-465** | *Bacillus cereus* group | Firmicutes, Bacilli, Bacillales, Bacillaceae | 5 | **+** | **+** | **-** | **-** | **-** | **-** | **-** | **+** |
| **L-578** | *Bacillus cereus* group | Firmicutes, Bacilli, Bacillales, Bacillaceae | 5 | **+** | **+** | **-** | **-** | **-** | **-** | **-** | **+** |
| **L-394** | *Bacillus cereus* group | Firmicutes, Bacilli, Bacillales, Bacillaceae | 6 | **+** | **+** | **-** | **-** | **-** | **-** | **-** | **+** |
| **L-839** | *Bacillus cereus* group | Firmicutes, Bacilli, Bacillales, Bacillaceae | 6 | **+** | **+** | **-** | **-** | **-** | **-** | **-** | **+** |
| **L-469** | *Bacillus cereus* group | Firmicutes, Bacilli, Bacillales, Bacillaceae | 6 | **+** | **+** | **-** | **-** | **+** | **-** | **-** | **+** |
| **L-755** | *Bacillus cereus* group | Firmicutes, Bacilli, Bacillales, Bacillaceae | 8 | **+** | **+** | **-** | **-** | **-** | **-** | **-** | **+** |
| **L-1078** | *Bacillus cereus* group | Firmicutes, Bacilli, Bacillales, Bacillaceae | 8 | **+** | **+** | **-** | **-** | **-** | **-** | **-** | **+** |
| **L-463** | *Bacillus cereus* group | Firmicutes, Bacilli, Bacillales, Bacillaceae | 8 | **+** | **+** | **-** | **-** | **-** | **-** | **-** | **+** |
| **L-756** | *Bacillus cereus* group | Firmicutes, Bacilli, Bacillales, Bacillaceae | 8 | **+** | **+** | **-** | **-** | **-** | **-** | **-** | **+** |
| **L-845** | *Bacillus cereus* group | Firmicutes, Bacilli, Bacillales, Bacillaceae | 9 | **+** | **+** | **-** | **-** | **-** | **-** | **-** | **+** |
| **L-846** | *Bacillus cereus* group | Firmicutes, Bacilli, Bacillales, Bacillaceae | 9 | **+** | **+** | **-** | **-** | **-** | **-** | **-** | **+** |
| **L-849** | *Bacillus cereus* group | Firmicutes, Bacilli, Bacillales, Bacillaceae | 9 | **+** | **+** | **-** | **-** | **-** | **-** | **-** | **+** |
| **L-911** | *Bacillus cereus* group | Firmicutes, Bacilli, Bacillales, Bacillaceae | 9 | **+** | **+** | **-** | **-** | **-** | **-** | **-** | **+** |
| **L-1037** | *Bacillus cereus* group | Firmicutes, Bacilli, Bacillales, Bacillaceae | 9 | **+** | **+** | **-** | **-** | **-** | **-** | **-** | **+** |
| **L-827** | *Bacillus circulans* | Firmicutes, Bacilli, Bacillales, Bacillaceae | 20 | **-** | **+** | **-** | **-** | **-** | **-** | **-** | **+** |
| **L-1000** | *Bacillus circulans* | Firmicutes, Bacilli, Bacillales, Bacillaceae | 20 | **-** | **+** | **-** | **-** | **-** |  | **-** | **+** |
| **L-672** | *Bacillus circulans* | Firmicutes, Bacilli, Bacillales, Bacillaceae | 20 | **-** | **+** | **-** | **-** | **-** | **-** | **-** | **+** |
| **L-1039** | *Bacillus firmus* | Firmicutes, Bacilli, Bacillales, Bacillaceae | 9 | **+** | **+** | **-** | **-** | **-** | **-** | **-** | **+** |
| **L-984** | *Bacillus flexus* | Firmicutes, Bacilli, Bacillales, Bacillaceae | 10 | **-** | **-** | **-** | **-** | **-** | **-** | **-** | **+** |
| **L-579** | *Bacillus flexus* | Firmicutes, Bacilli, Bacillales, Bacillaceae | 10 | **-** | **-** | **-** | **-** | **-** | **-** | **-** | **+** |
| **L-709** | *Bacillus flexus* | Firmicutes, Bacilli, Bacillales, Bacillaceae | 10 | **-** | **-** | **-** | **-** | **-** | **-** | **-** | **+** |
| **L-710** | *Bacillus flexus* | Firmicutes, Bacilli, Bacillales, Bacillaceae | 10 | **-** | **-** | **-** | **-** | **-** | **-** | **-** | **+** |
| **L-727** | *Bacillus flexus* | Firmicutes, Bacilli, Bacillales, Bacillaceae | 10 | **-** | **-** | **-** | **-** | **-** | **-** | **-** | **+** |
| **L-1055** | *Bacillus flexus* | Firmicutes, Bacilli, Bacillales, Bacillaceae | 10 | **-** | **-** | **-** | **-** | **-** | **-** | **-** | **+** |
| **L-1056** | *Bacillus flexus* | Firmicutes, Bacilli, Bacillales, Bacillaceae | 10 | **-** | **-** | **-** | **-** | **-** | **-** | **-** | **+** |
| **L-828** | *Bacillus flexus* | Firmicutes, Bacilli, Bacillales, Bacillaceae | 10 | **-** | **-** | **-** | **-** | **-** | **-** | **-** | **+** |
| **L-776** | *Bacillus flexus* | Firmicutes, Bacilli, Bacillales, Bacillaceae | 11 | **-** | **-** | **-** | **-** | **-** | **-** | **-** | **+** |
| **L-820** | *Bacillus flexus* | Firmicutes, Bacilli, Bacillales, Bacillaceae | 19 | **-** | **-** | **-** | **-** | **-** | **-** | **-** | **+** |
| **L-474** | *Bacillus flexus* | Firmicutes, Bacilli, Bacillales, Bacillaceae | 19 | **-** | **-** | **-** | **-** | **-** | **-** | **-** | **+** |
| **L-641** | *Bacillus flexus* | Firmicutes, Bacilli, Bacillales, Bacillaceae | 20 | **-** | **+** | **-** | **-** | **-** | **-** | **-** | **+** |
| **L-473** | *Bacillus flexus* | Firmicutes, Bacilli, Bacillales, Bacillaceae | 20 | **+** | **+** | **-** | **-** | **-** | **-** | **-** | **+** |
| **L-475** | *Bacillus flexus* | Firmicutes, Bacilli, Bacillales, Bacillaceae | 20 | **+** | **+** | **-** | **-** | **-** | **-** | **-** | **+** |
| **L-477** | *Bacillus flexus* | Firmicutes, Bacilli, Bacillales, Bacillaceae | 20 | **+** | **+** | **-** | **-** | **-** | **-** | **-** | **+** |
| **L-476** | *Bacillus flexus* | Firmicutes, Bacilli, Bacillales, Bacillaceae | 21 | **+** | **+** | **-** | **-** | **-** | **-** | **-** | **+** |
| **L-478** | *Bacillus flexus* | Firmicutes, Bacilli, Bacillales, Bacillaceae | 21 | **+** | **+** | **-** | **-** | **-** | **-** | **-** | **+** |
| **L-479** | *Bacillus flexus* | Firmicutes, Bacilli, Bacillales, Bacillaceae | 21 | **+** | **+** | **-** | **-** | **-** | **-** | **-** | **+** |
| **L-480** | *Bacillus flexus* | Firmicutes, Bacilli, Bacillales, Bacillaceae | 21 | **+** | **+** | **-** | **-** | **-** | **-** | **-** | **+** |
| **L-481** | *Bacillus flexus* | Firmicutes, Bacilli, Bacillales, Bacillaceae | 21 | **+** | **+** | **-** | **-** | **-** | **-** | **-** | **+** |
| **L-740** | *Bacillus flexus* | Firmicutes, Bacilli, Bacillales, Bacillaceae | 21 | **-** | **-** | **-** | **-** | **-** | **-** | **-** | **+** |
| **L-951** | *Bacillus flexus* | Firmicutes, Bacilli, Bacillales, Bacillaceae | 23 | **-** | **+** | **-** | **-** | **-** | **-** | **+** | **+** |
| **L-682** | *Bacillus flexus* | Firmicutes, Bacilli, Bacillales, Bacillaceae | 24 | **-** | **+** | **-** | **-** | **-** | **-** | **-** | **+** |
| **L-758** | *Bacillus flexus* | Firmicutes, Bacilli, Bacillales, Bacillaceae | 28 | **-** | **-** | **-** | **-** | **-** | **-** | **-** | **+** |
| **L-631** | *Bacillus flexus* | Firmicutes, Bacilli, Bacillales, Bacillaceae | 3 | **-** | **+** | **-** | **-** | **-** | **-** | **-** | **+** |
| **L-482** | *Bacillus flexus* | Firmicutes, Bacilli, Bacillales, Bacillaceae | 3 | **-** | **-** | **-** | **-** | **-** | **-** | **-** | **+** |
| **L-1049** | *Bacillus flexus* | Firmicutes, Bacilli, Bacillales, Bacillaceae | 31 | **-** | **-** | **-** | **-** | **-** | **-** | **-** | **+** |
| **L-647** | *Bacillus flexus* | Firmicutes, Bacilli, Bacillales, Bacillaceae | 34 | **-** | **+** | **-** | **-** | **-** | **-** | **-** | **+** |
| **L-1052** | *Bacillus flexus* | Firmicutes, Bacilli, Bacillales, Bacillaceae | 36 | **-** | **-** | **-** | **-** | **-** | **-** | **-** | **+** |
| **L-718** | *Bacillus flexus* | Firmicutes, Bacilli, Bacillales, Bacillaceae | 6 | **+** | **+** | **-** | **-** | **-** | **-** | **-** | **+** |
| **L-719** | *Bacillus flexus* | Firmicutes, Bacilli, Bacillales, Bacillaceae | 6 | **-** | **-** | **-** | **-** | **-** | **-** | **-** | **+** |
| **L-737** | *Bacillus flexus* | Firmicutes, Bacilli, Bacillales, Bacillaceae | 6 | **-** | **-** | **-** | **-** | **-** | **-** | **-** | **+** |
| **L-799** | *Bacillus flexus* | Firmicutes, Bacilli, Bacillales, Bacillaceae | 7 | **-** | **-** | **-** | **-** | **-** | **-** | **-** | **+** |
| **L-483** | *Bacillus flexus* | Firmicutes, Bacilli, Bacillales, Bacillaceae | 7 | **-** | **+** | **-** | **-** | **-** | **-** | **-** | **+** |
| **L-630** | *Bacillus flexus* | Firmicutes, Bacilli, Bacillales, Bacillaceae | 7 | **-** | **+** | **-** | **-** | **-** | **-** | **-** | **+** |
| **L-1032** | *Bacillus flexus* | Firmicutes, Bacilli, Bacillales, Bacillaceae | 9 | **+** | **+** | **-** | **-** | **-** | **-** | **-** | **+** |
| **L-1034** | *Bacillus flexus* | Firmicutes, Bacilli, Bacillales, Bacillaceae | 9 | **-** | **-** | **-** | **-** | **-** | **-** | **-** | **+** |
| **L-1038** | *Bacillus flexus* | Firmicutes, Bacilli, Bacillales, Bacillaceae | 9 | **-** | **-** | **-** | **-** | **-** | **-** | **-** | **+** |
| **L-1036** | *Bacillus flexus* | Firmicutes, Bacilli, Bacillales, Bacillaceae | 9 | **-** | **-** | **-** | **-** | **-** | **-** | **-** | **+** |
| **L-803** | *Bacillus horneckiae* | Firmicutes, Bacilli, Bacillales, Bacillaceae | 9 | **+** | **+** | **+** | **+** | **-** | **-** | **-** | **+** |
| **L-809** | *Bacillus horneckiae* | Firmicutes, Bacilli, Bacillales, Bacillaceae | 5 | **+** | **+** | **+** | **+** | **+** | **+** | **+** | **+** |
| **L-484** | *Bacillus licheniformis* | Firmicutes, Bacilli, Bacillales, Bacillaceae | 10 | **+** | **+** | **-** | **-** | **-** | **-** | **-** | **+** |
| **L-922** | *Bacillus pumilus* | Firmicutes, Bacilli, Bacillales, Bacillaceae | 11 | **+** | **+** | **-** | **-** | **-** | **-** | **-** | **+** |
| **L-485** | *Bacillus pumilus* | Firmicutes, Bacilli, Bacillales, Bacillaceae | 19 | **+** | **+** | **-** | **-** | **-** | **-** | **-** | **+** |
| **L-690** | *Bacillus pumilus* | Firmicutes, Bacilli, Bacillales, Bacillaceae | 19 | **+** | **+** | **+** | **-** | **-** | **-** | **-** | **+** |
| **L-691** | *Bacillus pumilus* | Firmicutes, Bacilli, Bacillales, Bacillaceae | 19 | **+** | **+** | **+** | **-** | **-** | **-** | **-** | **+** |
| **L-695** | *Bacillus pumilus* | Firmicutes, Bacilli, Bacillales, Bacillaceae | 19 | **+** | **+** | **+** | **-** | **-** | **-** | **-** | **+** |
| **L-580** | *Bacillus pumilus* | Firmicutes, Bacilli, Bacillales, Bacillaceae | 20 | **+** | **+** | **-** | **-** | **+** | **-** | **-** | **+** |
| **L-581** | *Bacillus pumilus* | Firmicutes, Bacilli, Bacillales, Bacillaceae | 20 | **+** | **+** | **-** | **-** | **+** | **-** | **-** | **+** |
| **L-583** | *Bacillus pumilus* | Firmicutes, Bacilli, Bacillales, Bacillaceae | 29 | **+** | **+** | **-** | **-** | **+** | **-** | **-** | **+** |
| **L-582** | *Bacillus pumilus* | Firmicutes, Bacilli, Bacillales, Bacillaceae | 30 | **+** | **+** | **-** | **-** | **+** | **-** | **-** | **+** |
| **L-1105** | *Bacillus pumilus* | Firmicutes, Bacilli, Bacillales, Bacillaceae | 9 | **+** | **+** | **-** | **-** | **-** | **-** | **-** | **+** |
| **L-1033** | *Bacillus pumilus* | Firmicutes, Bacilli, Bacillales, Bacillaceae | 9 | **+** | **+** | **-** | **-** | **-** | **-** | **-** | **+** |
| **L-1040** | *Bacillus pumilus* | Firmicutes, Bacilli, Bacillales, Bacillaceae | 9 | **+** | **+** | **-** | **-** | **-** | **-** | **-** | **+** |
| **L-486** | *Bacillus safensis* | Firmicutes, Bacilli, Bacillales, Bacillaceae | 19 | **+** | **+** | **-** | **-** | **-** | **-** | **-** | **+** |
| **L-487** | *Bacillus safensis* | Firmicutes, Bacilli, Bacillales, Bacillaceae | 19 | **+** | **+** | **-** | **-** | **-** | **-** | **-** | **+** |
| **L-689** | *Bacillus safensis* | Firmicutes, Bacilli, Bacillales, Bacillaceae | 19 | **+** | **+** | **-** | **-** | **-** | **-** | **-** | **+** |
| **L-696** | *Bacillus safensis* | Firmicutes, Bacilli, Bacillales, Bacillaceae | 19 | **+** | **+** | **-** | **-** | **-** | **-** | **-** | **+** |
| **L-850** | *Bacillus safensis* | Firmicutes, Bacilli, Bacillales, Bacillaceae | 29 | **+** | **+** | **-** | **-** | **-** | **-** | **-** | **+** |
| **L-1068** | *Bacillus safensis* | Firmicutes, Bacilli, Bacillales, Bacillaceae | 6 | **+** | **+** | **-** | **-** | **-** | **-** | **-** | **+** |
| **L-493** | *Bacillus* sp. | Firmicutes, Bacilli, Bacillales, Bacillaceae | 1 | **+** | **+** | **-** | **-** | **-** | **-** | **-** | **+** |
| **L-399** | *Bacillus* sp. | Firmicutes, Bacilli, Bacillales, Bacillaceae | 10 | **+** | **-** | **-** | **-** | **-** | **-** | **-** | **+** |
| **L-400** | *Bacillus* sp. | Firmicutes, Bacilli, Bacillales, Bacillaceae | 10 | **-** | **-** | **-** | **-** | **-** | **-** | **-** | **+** |
| **L-401** | *Bacillus* sp. | Firmicutes, Bacilli, Bacillales, Bacillaceae | 10 | **-** | **-** | **-** | **-** | **-** | **-** | **-** | **+** |
| **L-403** | *Bacillus* sp. | Firmicutes, Bacilli, Bacillales, Bacillaceae | 10 | **-** | **-** | **-** | **-** | **-** | **-** | **-** | **+** |
| **L-770** | *Bacillus* sp. | Firmicutes, Bacilli, Bacillales, Bacillaceae | 11 | **+** | **+** | **-** | **-** | **-** | **-** | **-** | **+** |
| **L-775** | *Bacillus* sp. | Firmicutes, Bacilli, Bacillales, Bacillaceae | 11 | **+** | **+** | **-** | **-** | **-** | **-** | **-** | **+** |
| **L-489** | *Bacillus* sp. | Firmicutes, Bacilli, Bacillales, Bacillaceae | 19 | **+** | **+** | **-** | **-** | **-** | **-** | **-** | **+** |
| **L-584** | *Bacillus* sp. | Firmicutes, Bacilli, Bacillales, Bacillaceae | 19 | **+** | **+** | **-** | **-** | **-** | **-** | **-** | **+** |
| **L-639** | *Bacillus* sp. | Firmicutes, Bacilli, Bacillales, Bacillaceae | 19 | **-** | **-** | **-** | **-** | **-** | **-** | **-** | **+** |
| **L-494** | *Bacillus* sp. | Firmicutes, Bacilli, Bacillales, Bacillaceae | 2 | **+** | **+** | **-** | **-** | **-** | **-** | **-** | **+** |
| **L-492** | *Bacillus* sp. | Firmicutes, Bacilli, Bacillales, Bacillaceae | 21 | **+** | **+** | **-** | **-** | **-** | **-** | **-** | **+** |
| **L-502** | *Bacillus* sp. | Firmicutes, Bacilli, Bacillales, Bacillaceae | 23 | **+** | **+** | **-** | **-** | **-** | **-** | **-** | **+** |
| **L-844** | *Bacillus* sp*.* | Firmicutes, Bacilli, Bacillales, Bacillaceae | 27 | **+** | **+** | **-** | **-** | **-** | **-** | **-** | **+** |
| **L-497** | *Bacillus* sp. | Firmicutes, Bacilli, Bacillales, Bacillaceae | 29 | **-** | **+** | **-** | **-** | **-** | **-** | **-** | **+** |
| **L-503** | *Bacillus* sp. | Firmicutes, Bacilli, Bacillales, Bacillaceae | 3 | **-** | **+** | **-** | **-** | **-** | **-** | **-** | **+** |
| **L-504** | *Bacillus* sp. | Firmicutes, Bacilli, Bacillales, Bacillaceae | 3 | **-** | **+** | **-** | **-** | **-** | **-** | **-** | **+** |
| **L-505** | *Bacillus* sp. | Firmicutes, Bacilli, Bacillales, Bacillaceae | 3 | **-** | **+** | **-** | **-** | **-** | **-** | **-** | **+** |
| **L-852** | *Bacillus* sp. | Firmicutes, Bacilli, Bacillales, Bacillaceae | 32 | **+** | **+** | **-** | **-** | **-** | **-** | **-** | **+** |
| **L-853** | *Bacillus* sp. | Firmicutes, Bacilli, Bacillales, Bacillaceae | 32 | **+** | **+** | **-** | **-** | **-** | **-** | **-** | **+** |
| **L-495** | *Bacillus* sp. | Firmicutes, Bacilli, Bacillales, Bacillaceae | 4 | **-** | **+** | **-** | **-** | **-** | **-** | **-** | **+** |
| **L-496** | *Bacillus* sp. | Firmicutes, Bacilli, Bacillales, Bacillaceae | 4 | **-** | **+** | **-** | **-** | **-** | **-** | **-** | **+** |
| **L-501** | *Bacillus* sp. | Firmicutes, Bacilli, Bacillales, Bacillaceae | 4 | **+** | **+** | **-** | **-** | **-** | **-** | **-** | **+** |
| **L-507** | *Bacillus* sp. | Firmicutes, Bacilli, Bacillales, Bacillaceae | 4 | **-** | **-** | **-** | **-** | **-** | **-** | **-** | **+** |
| **L-508** | *Bacillus* sp. | Firmicutes, Bacilli, Bacillales, Bacillaceae | 4 | **-** | **+** | **-** | **-** | **-** | **-** | **-** | **+** |
| **L-509** | *Bacillus* sp. | Firmicutes, Bacilli, Bacillales, Bacillaceae | 4 | **-** | **+** | **-** | **-** | **-** | **-** | **-** | **+** |
| **L-380** | *Bacillus* sp. | Firmicutes, Bacilli, Bacillales, Bacillaceae | 4 | **+** | **+** | **-** | **-** | **-** | **-** | **-** | **+** |
| **L-498** | *Bacillus* sp. | Firmicutes, Bacilli, Bacillales, Bacillaceae | 7 | **-** | **-** | **-** | **-** | **-** | **-** | **-** | **+** |
| **L-499** | *Bacillus* sp. | Firmicutes, Bacilli, Bacillales, Bacillaceae | 7 | **-** | **+** | **-** | **-** | **-** | **-** | **-** | **+** |
| **L-500** | *Bacillus* sp. | Firmicutes, Bacilli, Bacillales, Bacillaceae | 7 | **-** | **-** | **-** | **-** | **-** | **-** | **-** | **+** |
| **L-1035** | *Bacillus* sp. | Firmicutes, Bacilli, Bacillales, Bacillaceae | 9 | **+** | **+** | **+** | **+** | **-** | **-** | **-** | **+** |
| **L-773** | *Bacillus subtilis* | Firmicutes, Bacilli, Bacillales, Bacillaceae | 11 | **-** | **+** | **-** | **-** | **-** | **-** | **-** | **+** |
| **L-511** | *Bacillus subtilis* | Firmicutes, Bacilli, Bacillales, Bacillaceae | 21 | **+** | **+** | **-** | **-** | **-** | **-** | **-** | **+** |
| **L-793** | *Bacillus subtilis* | Firmicutes, Bacilli, Bacillales, Bacillaceae | 27 | **+** | **+** | **-** | **-** | **-** | **-** | **-** | **+** |
| **L-910** | *Bacillus subtilis* | Firmicutes, Bacilli, Bacillales, Bacillaceae | 28 | **+** | **+** | **-** | **-** | **-** | **-** | **-** | **+** |
| **L-512** | *Bacillus subtilis* | Firmicutes, Bacilli, Bacillales, Bacillaceae | 28 | **+** | **+** | **-** | **-** | **-** | **-** | **-** | **+** |
| **L-513** | *Bacillus subtilis* | Firmicutes, Bacilli, Bacillales, Bacillaceae | 28 | **+** | **+** | **-** | **-** | **-** | **-** | **-** | **+** |
| **L-514** | *Bacillus subtilis* | Firmicutes, Bacilli, Bacillales, Bacillaceae | 28 | **+** | **+** | **-** | **-** | **-** | **-** | **-** | **+** |
| **L-904** | *Bacillus subtilis* | Firmicutes, Bacilli, Bacillales, Bacillaceae | 28 | **+** | **+** | **+** | **-** | **-** | **-** | **-** | **++** |
| **L-905** | *Bacillus subtilis* | Firmicutes, Bacilli, Bacillales, Bacillaceae | 28 | **+** | **+** | **+** | **-** | **-** | **-** | **-** | **+** |
| **L-515** | *Bacillus subtilis* | Firmicutes, Bacilli, Bacillales, Bacillaceae | 29 | **+** | **+** | **-** | **-** | **-** | **-** | **-** | **+** |
| **L-518** | *Bacillus subtilis* | Firmicutes, Bacilli, Bacillales, Bacillaceae | 29 | **+** | **-** | **-** | **-** | **-** | **-** | **-** | **+** |
| **L-519** | *Bacillus subtilis* | Firmicutes, Bacilli, Bacillales, Bacillaceae | 29 | **+** | **+** | **-** | **-** | **-** | **-** | **-** | **+** |
| **L-516** | *Bacillus subtilis* | Firmicutes, Bacilli, Bacillales, Bacillaceae | 3 | **+** | **+** | **-** | **-** | **-** | **-** | **-** | **+** |
| **L-523** | *Bacillus subtilis* | Firmicutes, Bacilli, Bacillales, Bacillaceae | 3 | **+** | **+** | **-** | **-** | **-** | **-** | **-** | **+** |
| **L-524** | *Bacillus subtilis* | Firmicutes, Bacilli, Bacillales, Bacillaceae | 3 | **+** | **+** | **-** | **-** | **-** | **-** | **-** | **+** |
| **L-517** | *Bacillus subtilis* | Firmicutes, Bacilli, Bacillales, Bacillaceae | 30 | **+** | **+** | **-** | **-** | **-** | **-** | **-** | **+** |
| **L-794** | *Bacillus subtilis* | Firmicutes, Bacilli, Bacillales, Bacillaceae | 30 | **+** | **+** | **-** | **-** | **-** | **-** | **-** | **+** |
| **L-795** | *Bacillus subtilis* | Firmicutes, Bacilli, Bacillales, Bacillaceae | 30 | **+** | **+** | **-** | **-** | **-** | **-** | **-** | **+** |
| **L-907** | *Bacillus subtilis* | Firmicutes, Bacilli, Bacillales, Bacillaceae | 30 | **+** | **+** | **-** | **-** | **-** | **-** | **-** | **+** |
| **L-1077** | *Bacillus subtilis* | Firmicutes, Bacilli, Bacillales, Bacillaceae | 31 | **-** | **+** | **-** | **-** | **-** | **-** | **-** | **+** |
| **L-797** | *Bacillus subtilis* | Firmicutes, Bacilli, Bacillales, Bacillaceae | 34 | **-** | **-** | **-** | **-** | **-** | **-** | **-** | **+** |
| **L-510** | *Bacillus subtilis* | Firmicutes, Bacilli, Bacillales, Bacillaceae | 4 | **-** | **+** | **-** | **-** | **-** | **-** | **-** | **+** |
| **L-586** | *Bacillus subtilis* | Firmicutes, Bacilli, Bacillales, Bacillaceae | 5 | **+** | **+** | **-** | **-** | **-** | **-** | **-** | **+** |
| **L-585** | *Bacillus subtilis* | Firmicutes, Bacilli, Bacillales, Bacillaceae | 6 | **+** | **+** | **-** | **-** | **-** | **-** | **-** | **+** |
| **L-865** | *Bacillus subtilis* | Firmicutes, Bacilli, Bacillales, Bacillaceae | 8 | **+** | **+** | **-** | **-** | **-** | **-** | **-** | **+** |
| **L-520** | *Bacillus subtilis* | Firmicutes, Bacilli, Bacillales, Bacillaceae | 8 | **-** | **-** | **-** | **-** | **-** | **-** | **-** | **+** |
| **L-521** | *Bacillus subtilis* | Firmicutes, Bacilli, Bacillales, Bacillaceae | 8 | **+** | **+** | **-** | **-** | **-** | **-** | **-** | **+** |
| **L-522** | *Bacillus subtilis* | Firmicutes, Bacilli, Bacillales, Bacillaceae | 8 | **+** | **+** | **-** | **-** | **-** | **-** | **-** | **+** |
| **L-976** | *Brachybacterium* sp. | Actinobacteria, Actinobacteria, Actinomycetales, Dermabacteraceae | 29 | **-** | **+** | **+** | **+** | **+** | **-** | **-** | **+** |
| **L-408** | *Brevibacillus parabrevis* | Firmicutes, Bacilli, Bacillales, Paenibacillaceae 1 | 4 | **-** | **-** | **-** | **-** | **-** | **-** | **-** | **+** |
| **L-415** | *Brevibacillus parabrevis* | Firmicutes, Bacilli, Bacillales, Paenibacillaceae 1 | 10 | **-** | **-** | **-** | **-** | **-** | **-** | **-** | **+** |
| **L-798** | *Brevibacillus parabrevis* | Firmicutes, Bacilli, Bacillales, Paenibacillaceae 1 | 10 | **-** | **-** | **-** | **-** | **-** | **-** | **-** | **+** |
| **L-866** | *Brevibacterium casei* | Actinobacteria, Actinobacteria, Actinomycetales, Brevibacteriaceae | 15 | **+** | **+** | **+** | **+** | **+** | **-** | **-** | **+** |
| **L-955** | *Brevibacterium casei* | Actinobacteria, Actinobacteria, Actinomycetales, Micrococcineae | 15 | **+** | **+** | **+** | **+** | **+** | **-** | **-** | **+** |
| **L-957** | *Brevibacterium casei* | Actinobacteria, Actinobacteria, Actinomycetales, Micrococcineae | 15 | **+** | **+** | **+** | **+** | **+** | **-** | **-** | **+** |
| **L-1028** | *Brevibacterium casei* | Actinobacteria, Actinobacteria, Actinomycetales, Brevibacteriaceae | 22 | **+** | **+** | **+** | **+** | **+** | **-** | **-** | **+** |
| **L-1029** | *Brevibacterium casei* | Actinobacteria, Actinobacteria, Actinomycetales, Brevibacteriaceae | 22 | **+** | **+** | **+** | **+** | **+** | **-** | **-** | **+** |
| **L-943** | *Brevibacterium casei* | Actinobacteria, Actinobacteria, Actinomycetales, Micrococcineae | 22 | **+** | **+** | **+** | **+** | **+** | **-** | **-** | **+** |
| **L-942** | *Brevibacterium casei* | Actinobacteria, Actinobacteria, Actinomycetales, Micrococcineae | 25 | **+** | **+** | **+** | **+** | **+** | **-** | **-** | **+** |
| **L-977** | *Brevibacterium casei* | Actinobacteria, Actinobacteria, Actinomycetales, Micrococcineae | 27 | **-** | **+** | **+** | **-** | **-** | **-** | **-** | **+** |
| **L-663** | *Brevibacterium casei* | Actinobacteria, Actinobacteria, Actinomycetales, Micrococcineae | 33 | **-** | **+** | **+** | **+** | **+** | **-** | **-** | **+** |
| **L-645** | *Brevibacterium sanguinus* | Actinobacteria, Actinobacteria, Actinomycetales, Micrococcineae | 33 | **-** | **+** | **+** | **+** | **+** | **-** | **-** | **+** |
| **L-662** | *Brevibacterium sanguinus* | Actinobacteria, Actinobacteria, Actinomycetales, Micrococcineae | 33 | **-** | **-** | **+** | **+** | **+** | **-** | **-** | **+** |
| **L-664** | *Brevibacterium sanguinus* | Actinobacteria, Actinobacteria, Actinomycetales, Micrococcineae | 33 | **-** | **+** | **+** | **+** | **+** | **-** | **-** | **+** |
| **L-867** | *Brevundimonas diminuta* | Proteobacteria, Alphaproteobacteria, Caulobacterales, Caulobacteraceae | 24 | **+** | **+** | **+** | **+** | **+** | **-** | **-** | **+** |
| **L-1047** | *Cellulosimicrobium cellulans* | Actinobacteria, Actinobacteria, Actinomycetales, Promicromonosporaceae | 31 | **+** | **+** | **+** | **+** | **+** | **+** | **+** | **+** |
| **L-869** | *Cellulosimicrobium cellulans* | Actinobacteria, Actinobacteria, Actinomycetales, Promicromonosporaceae | 24 | **+** | **+** | **+** | **+** | **+** | **-** | **+** | **+** |
| **L-872** | *Cellulosimicrobium cellulans* | Actinobacteria, Actinobacteria, Actinomycetales, Promicromonosporaceae | 24 | **+** | **+** | **+** | **+** | **+** | **-** | **+** | **+** |
| **L-873** | *Cellulosimicrobium cellulans* | Actinobacteria, Actinobacteria, Actinomycetales, Promicromonosporaceae | 24 | **+** | **+** | **+** | **+** | **+** | **-** | **+** | **+** |
| **L-941** | *Cellulosimicrobium cellulans* | Actinobacteria, Actinobacteria, Actinomycetales, Promicromonosporaceae | 25 | **+** | **+** | **+** | **+** | **+** | **-** | **+** | **+** |
| **L-536** | *Chryseobacterium* sp. | Bacteroidetes, Flavobacteria, Flavobacteriales, Flavobacteriaceae | 10 | **+** | **+** | **+** | **+** | **+** | **+** | **+** | **+** |
| **L-532** | *Chryseobacterium* sp. | Bacteroidetes, Flavobacteria, Flavobacteriales, Flavobacteriaceae | 2 | **+** | **+** | **+** | **+** | **+** | **+** | **+** | **+** |
| **L-533** | *Chryseobacterium* sp. | Bacteroidetes, Flavobacteria, Flavobacteriales, Flavobacteriaceae | 2 | **+** | **+** | **+** | **+** | **+** | **+** | **+** | **+** |
| **L-534** | *Chryseobacterium* sp. | Bacteroidetes, Flavobacteria, Flavobacteriales, Flavobacteriaceae | 2 | **+** | **+** | **+** | **+** | **+** | **+** | **+** | **+** |
| **L-535-1** | *Chryseobacterium* sp. | Bacteroidetes, Flavobacteria, Flavobacteriales, Flavobacteriaceae | 2 | **+** | **-** | **+** | **+** | **+** | **+** | **+** | **+** |
| **L-535-2** | *Chryseobacterium* sp. | Bacteroidetes, Flavobacteria, Flavobacteriales, Flavobacteriaceae | 2 | **+** | **-** | **+** | **+** | **+** | **+** | **+** | **+** |
| **L-537** | *Chryseobacterium* sp. | Bacteroidetes, Flavobacteria, Flavobacteriales, Flavobacteriaceae | 2 | **+** | **+** | **+** | **+** | **+** | **+** | **+** | **+** |
| **L-538** | *Chryseobacterium* sp. | Bacteroidetes, Flavobacteria, Flavobacteriales, Flavobacteriaceae | 2 | **+** | **-** | **+** | **+** | **+** | **+** | **+** | **+** |
| **L-657** | *Chryseobacterium* sp. | Bacteroidetes, Flavobacteria, Flavobacteriales, Flavobacteriaceae | 2 | **+** | **-** | **+** | **+** | **+** | **+** | **+** | **+** |
| **L-678** | *Chryseobacterium* sp. | Bacteroidetes, Flavobacteria, Flavobacteriales, Flavobacteriaceae | 2 | **-** | **-** | **+** | **+** | **+** | **-** | **+** | **+** |
| **L-700** | *Chryseobacterium* sp. | Bacteroidetes, Flavobacteria, Flavobacteriales, Flavobacteriaceae | 2 | **+** | **+** | **+** | **+** | **+** | **+** | **+** | **+** |
| **L-705** | *Chryseobacterium* sp. | Bacteroidetes, Flavobacteria, Flavobacteriales, Flavobacteriaceae | 2 | **+** | **-** | **+** | **+** | **+** | **+** | **+** | **+** |
| **L-749** | *Chryseobacterium* sp. | Bacteroidetes, Flavobacteria, Flavobacteriales, Flavobacteriaceae | 2 | **+** | **+** | **+** | **+** | **+** | **+** | **+** | **+** |
| **L-751** | *Chryseobacterium* sp. | Bacteroidetes, Flavobacteria, Flavobacteriales, Flavobacteriaceae | 2 | **+** | **+** | **+** | **+** | **+** | **+** | **+** | **+** |
| **L-959** | *Chryseobacterium* sp. | Bacteroidetes, Flavobacteria, Flavobacteriales, Flavobacteriaceae | 2 | **+** | **+** | **+** | **+** | **+** | **+** | **+** | **+** |
| **L-539** | *Chryseobacterium* sp. | Bacteroidetes, Flavobacteria, Flavobacteriales, Flavobacteriaceae | 2 | **+** | **+** | **+** | **-** | **+** | **-** | **+** | **+** |
| **L-531** | *Chryseobacterium* sp. | Bacteroidetes, Flavobacteria, Flavobacteriales, Flavobacteriaceae | 4 | **+** | **+** | **+** | **+** | **+** | **+** | **+** | **+** |
| **L-409** | *Chryseobacterium* sp. | Bacteroidetes, Flavobacteria, Flavobacteriales, Flavobacteriaceae | 6 | **+** | **+** | **+** | **+** | **+** | **+** | **+** | **+** |
| **L-1732** | *Clostridium xylanolyticum* | Firmicutes, Clostridia, Clostridiales, Clostridiaceae | 10 | **-** | **-** | **-** | **-** | **-** | **-** | **-** | **-** |
| **L-1069** | *Comamonas aquatica* | Proteobacteria, Betaproteobacteria, Burkholderiales, Comamonadaceae | 6 | **+** | **+** | **-** | **-** | **-** | **-** | **-** | **+** |
| **L-874** | *Corynebacterium* sp. | Actinobacteria, Actinobacteria, Actinomycetales, Corynebacteriaceae | 23 | **-** | **-** | **-** | **-** | **-** | **-** | **-** | **+** |
| **L-791** | *Cronobacter sakazakii* | Proteobacteria, Gammaproteobacteria, Enterobacteriales, Enterobacteriaceae | 13 | **-** | **-** | **-** | **-** | **-** | **-** | **-** | **+** |
| **L-1010** | *Cronobacter sakazakii* | Proteobacteria, Gammaproteobacteria, Enterobacteriales, Enterobacteriaceae | 13 | **+** | **+** | **+** | **+** | **+** | **+** | **+** | **+** |
| **L-974** | *Cronobacter sakazakii* | Proteobacteria, Gammaproteobacteria, Enterobacteriales, Enterobacteriaceae | 27 | **+** | **+** | **+** | **+** | **+** | **-** | **-** | **+** |
| **L-1106** | *Enterobacter* sp. | Proteobacteria, Gammaproteobacteria, Enterobacteriales, Enterobacteriaceae | 12 | **-** | **+** | **-** | **+** | **-** | **-** | **-** | **+** |
| **L-540** | *Enterobacter* sp. | Proteobacteria, Gammaproteobacteria, Enterobacteriales, Enterobacteriaceae | 2 | **+** | **-** | **-** | **+** | **-** | **-** | **-** | **+** |
| **L-715** | *Enterobacter* sp. | Proteobacteria, Gammaproteobacteria, Enterobacteriales, Enterobacteriaceae | 2 | **-** | **-** | **+** | **+** | **-** | **-** | **-** | **+** |
| **L-385** | *Enterobacter* sp. | Proteobacteria, Gammaproteobacteria, Enterobacteriales, Enterobacteriaceae | 4 | **-** | **-** | **-** | **+** | **-** | **-** | **-** | **+** |
| **L-390** | *Enterobacter* sp. | Proteobacteria, Gammaproteobacteria, Enterobacteriales, Enterobacteriaceae | 6 | **+** | **-** | **-** | **+** | **-** | **-** | **-** | **+** |
| **L-393** | *Enterobacter* sp. | Proteobacteria, Gammaproteobacteria, Enterobacteriales, Enterobacteriaceae | 6 | **+** | **-** | **-** | **+** | **-** | **-** | **-** | **+** |
| **L-863** | *Enterobacter* sp. | Proteobacteria, Gammaproteobacteria, Enterobacteriales, Enterobacteriaceae | 6 | **-** | **-** | **-** | **+** | **-** | **-** | **-** | **+** |
| **L-944** | *Enterococcus casseliflavus* | Firmicutes, Bacilli, Lactobacillales, Enterococcaceae | 1 | **+** | **+** | **+** | **+** | **+** | **+** | **+** | **+** |
| **L-1067** | *Enterococcus casseliflavus* | Firmicutes, Bacilli, Lactobacillales, Enterococcaceae | 1 | **+** | **+** | **+** | **+** | **+** | **-** | **-** | **+** |
| **L-634** | *Enterococcus casseliflavus* | Firmicutes, Bacilli, Lactobacillales, Enterococcaceae | 2 | **+** | **+** | **+** | **-** | **+** | **-** | **+** | **+** |
| **L-824** | *Enterococcus casseliflavus* | Firmicutes, Bacilli, Lactobacillales, Enterococcaceae | 20 | **+** | **+** | **+** | **-** | **+** | **-** | **+** | **+** |
| **L-826** | *Enterococcus casseliflavus* | Firmicutes, Bacilli, Lactobacillales, Enterococcaceae | 20 | **+** | **+** | **+** | **-** | **+** | **-** | **+** | **+** |
| **L-780** | *Enterococcus casseliflavus* | Firmicutes, Bacilli, Lactobacillales, Enterococcaceae | 20 | **+** | **+** | **+** | **-** | **+** | **-** | **+** | **+** |
| **L-711** | *Enterococcus casseliflavus* | Firmicutes, Bacilli, Lactobacillales, Enterococcaceae | 20 | **+** | **+** | **+** | **-** | **+** | **-** | **+** | **+** |
| **L-543** | *Enterococcus casseliflavus* | Firmicutes, Bacilli, Lactobacillales, Enterococcaceae | 20 | **+** | **+** | **-** | **-** | **+** | **-** | **-** | **+** |
| **L-546** | *Enterococcus casseliflavus* | Firmicutes, Bacilli, Lactobacillales, Enterococcaceae | 20 | **+** | **+** | **+** | **-** | **+** | **-** | **+** | **+** |
| **L-547** | *Enterococcus casseliflavus* | Firmicutes, Bacilli, Lactobacillales, Enterococcaceae | 20 | **+** | **+** | **+** | **-** | **+** | **-** | **+** | **+** |
| **L-674** | *Enterococcus casseliflavus* | Firmicutes, Bacilli, Lactobacillales, Enterococcaceae | 20 | **+** | **+** | **+** | **-** | **-** | **-** | **-** | **+** |
| **L-676** | *Enterococcus casseliflavus* | Firmicutes, Bacilli, Lactobacillales, Enterococcaceae | 20 | **+** | **+** | **+** | **-** | **+** | **-** | **-** | **+** |
| **L-738** | *Enterococcus casseliflavus* | Firmicutes, Bacilli, Lactobacillales, Enterococcaceae | 20 | **+** | **+** | **+** | **-** | **+** | **-** | **-** | **+** |
| **L-777** | *Enterococcus casseliflavus* | Firmicutes, Bacilli, Lactobacillales, Enterococcaceae | 20 | **+** | **+** | **+** | **-** | **+** | **-** | **+** | **+** |
| **L-779** | *Enterococcus casseliflavus* | Firmicutes, Bacilli, Lactobacillales, Enterococcaceae | 20 | **+** | **+** | **+** | **-** | **+** | **-** | **+** | **+** |
| **L-946** | *Enterococcus casseliflavus* | Firmicutes, Bacilli, Lactobacillales, Enterococcaceae | 20 | **+** | **+** | **+** | **-** | **+** | **-** | **-** | **+** |
| **L-1060** | *Enterococcus casseliflavus* | Firmicutes, Bacilli, Lactobacillales, Enterococcaceae | 20 | **+** | **+** | **+** | **-** | **+** | **-** | **-** | **+** |
| **L-1102** | *Enterococcus casseliflavus* | Firmicutes, Bacilli, Lactobacillales, Enterococcaceae | 20 | **+** | **+** | **+** | **-** | **+** | **-** | **-** | **+** |
| **L-550** | *Enterococcus casseliflavus* | Firmicutes, Bacilli, Lactobacillales, Enterococcaceae | 20 | **+** | **+** | **+** | **-** | **+** | **-** | **+** | **+** |
| **L-551** | *Enterococcus casseliflavus* | Firmicutes, Bacilli, Lactobacillales, Enterococcaceae | 20 | **+** | **+** | **+** | **-** | **+** | **-** | **+** | **+** |
| **L-829** | *Enterococcus casseliflavus* | Firmicutes, Bacilli, Lactobacillales, Enterococcaceae | 21 | **+** | **+** | **+** | **-** | **+** | **-** | **+** | **+** |
| **L-882** | *Enterococcus casseliflavus* | Firmicutes, Bacilli, Lactobacillales, Enterococcaceae | 22 | **+** | **+** | **+** | **-** | **+** | **-** | **+** | **+** |
| **L-1062** | *Enterococcus casseliflavus* | Firmicutes, Bacilli, Lactobacillales, Enterococcaceae | 22 | **+** | **+** | **+** | **-** | **+** | **-** | **+** | **+** |
| **L-1096** | *Enterococcus casseliflavus* | Firmicutes, Bacilli, Lactobacillales, Enterococcaceae | 22 | **+** | **+** | **+** | **-** | **+** | **-** | **+** | **+** |
| **L-1097** | *Enterococcus casseliflavus* | Firmicutes, Bacilli, Lactobacillales, Enterococcaceae | 22 | **+** | **+** | **+** | **-** | **+** | **-** | **+** | **+** |
| **L-1098** | *Enterococcus casseliflavus* | Firmicutes, Bacilli, Lactobacillales, Enterococcaceae | 22 | **+** | **+** | **+** | **-** | **+** | **-** | **+** | **+** |
| **L-723** | *Enterococcus casseliflavus* | Firmicutes, Bacilli, Lactobacillales, Enterococcaceae | 22 | **+** | **-** | **+** | **-** | **-** | **-** | **-** | **+** |
| **L-892** | *Enterococcus casseliflavus* | Firmicutes, Bacilli, Lactobacillales, Enterococcaceae | 22 | **+** | **+** | **+** | **+** | **+** | **-** | **+** | **+** |
| **L-541** | *Enterococcus casseliflavus* | Firmicutes, Bacilli, Lactobacillales, Enterococcaceae | 24 | **+** | **+** | **+** | **-** | **+** | **-** | **+** | **+** |
| **L-542** | *Enterococcus casseliflavus* | Firmicutes, Bacilli, Lactobacillales, Enterococcaceae | 24 | **+** | **+** | **+** | **-** | **+** | **-** | **+** | **+** |
| **L-830** | *Enterococcus casseliflavus* | Firmicutes, Bacilli, Lactobacillales, Enterococcaceae | 24 | **+** | **+** | **+** | **-** | **+** | **-** | **+** | **+** |
| **L-831** | *Enterococcus casseliflavus* | Firmicutes, Bacilli, Lactobacillales, Enterococcaceae | 24 | **+** | **+** | **+** | **-** | **+** | **-** | **+** | **+** |
| **L-832** | *Enterococcus casseliflavus* | Firmicutes, Bacilli, Lactobacillales, Enterococcaceae | 24 | **+** | **+** | **+** | **-** | **+** | **-** | **+** | **+** |
| **L-833** | *Enterococcus casseliflavus* | Firmicutes, Bacilli, Lactobacillales, Enterococcaceae | 24 | **+** | **+** | **+** | **-** | **+** | **-** | **+** | **+** |
| **L-1094** | *Enterococcus casseliflavus* | Firmicutes, Bacilli, Lactobacillales, Enterococcaceae | 24 | **+** | **+** | **+** | **-** | **+** | **-** | **+** | **+** |
| **L-834** | *Enterococcus casseliflavus* | Firmicutes, Bacilli, Lactobacillales, Enterococcaceae | 24 | **-** | **+** | **+** | **-** | **+** | **-** | **+** | **+** |
| **L-544** | *Enterococcus casseliflavus* | Firmicutes, Bacilli, Lactobacillales, Enterococcaceae | 24 | **+** | **+** | **+** | **-** | **+** | **-** | **+** | **+** |
| **L-545** | *Enterococcus casseliflavus* | Firmicutes, Bacilli, Lactobacillales, Enterococcaceae | 24 | **+** | **+** | **+** | **-** | **+** | **-** | **+** | **+** |
| **L-587** | *Enterococcus casseliflavus* | Firmicutes, Bacilli, Lactobacillales, Enterococcaceae | 24 | **+** | **+** | **+** | **-** | **+** | **-** | **+** | **+** |
| **L-588** | *Enterococcus casseliflavus* | Firmicutes, Bacilli, Lactobacillales, Enterococcaceae | 24 | **+** | **+** | **+** | **-** | **+** | **-** | **+** | **+** |
| **L-680** | *Enterococcus casseliflavus* | Firmicutes, Bacilli, Lactobacillales, Enterococcaceae | 24 | **+** | **+** | **+** | **-** | **+** | **-** | **+** | **+** |
| **L-683** | *Enterococcus casseliflavus* | Firmicutes, Bacilli, Lactobacillales, Enterococcaceae | 24 | **+** | **+** | **+** | **-** | **+** | **-** | **+** | **+** |
| **L-712** | *Enterococcus casseliflavus* | Firmicutes, Bacilli, Lactobacillales, Enterococcaceae | 24 | **+** | **+** | **+** | **-** | **+** | **-** | **-** | **+** |
| **L-713** | *Enterococcus casseliflavus* | Firmicutes, Bacilli, Lactobacillales, Enterococcaceae | 24 | **+** | **+** | **+** | **-** | **+** | **-** | **-** | **+** |
| **L-885** | *Enterococcus casseliflavus* | Firmicutes, Bacilli, Lactobacillales, Enterococcaceae | 24 | **+** | **+** | **+** | **-** | **+** | **-** | **+** | **+** |
| **L-886** | *Enterococcus casseliflavus* | Firmicutes, Bacilli, Lactobacillales, Enterococcaceae | 24 | **+** | **+** | **+** | **-** | **+** | **-** | **+** | **+** |
| **L-887** | *Enterococcus casseliflavus* | Firmicutes, Bacilli, Lactobacillales, Enterococcaceae | 24 | **+** | **+** | **+** | **-** | **+** | **-** | **+** | **+** |
| **L-888** | *Enterococcus casseliflavus* | Firmicutes, Bacilli, Lactobacillales, Enterococcaceae | 24 | **+** | **+** | **+** | **-** | **+** | **-** | **+** | **+** |
| **L-1091** | *Enterococcus casseliflavus* | Firmicutes, Bacilli, Lactobacillales, Enterococcaceae | 24 | **+** | **+** | **+** | **-** | **-** | **-** | **-** | **+** |
| **L-1092** | *Enterococcus casseliflavus* | Firmicutes, Bacilli, Lactobacillales, Enterococcaceae | 24 | **+** | **+** | **+** | **-** | **+** | **-** | **+** | **+** |
| **L-1093** | *Enterococcus casseliflavus* | Firmicutes, Bacilli, Lactobacillales, Enterococcaceae | 24 | **+** | **+** | **+** | **-** | **+** | **-** | **+** | **+** |
| **L-1095** | *Enterococcus casseliflavus* | Firmicutes, Bacilli, Lactobacillales, Enterococcaceae | 24 | **+** | **+** | **+** | **-** | **+** | **-** | **+** | **+** |
| **L-900** | *Enterococcus casseliflavus* | Firmicutes, Bacilli, Lactobacillales, Enterococcaceae | 25 | **+** | **+** | **+** | **-** | **+** | **-** | **-** | **+** |
| **L-938** | *Enterococcus casseliflavus* | Firmicutes, Bacilli, Lactobacillales, Enterococcaceae | 25 | **+** | **+** | **+** | **-** | **+** | **-** | **-** | **+** |
| **L-754** | *Enterococcus casseliflavus* | Firmicutes, Bacilli, Lactobacillales, Enterococcaceae | 29 | **+** | **+** | **+** | **-** | **+** | **-** | **-** | **+** |
| **L-789** | *Enterococcus casseliflavus* | Firmicutes, Bacilli, Lactobacillales, Enterococcaceae | 6 | **-** | **+** | **+** | **-** | **-** | **-** | **-** | **+** |
| **L-729** | *Enterococcus casseliflavus* | Firmicutes, Bacilli, Lactobacillales, Enterococcaceae | 6 | **+** | **+** | **+** | **-** | **+** | **-** | **-** | **+** |
| **L-548** | *Enterococcus casseliflavus* | Firmicutes, Bacilli, Lactobacillales, Enterococcaceae | 6 | **+** | **+** | **+** | **-** | **+** | **-** | **+** | **+** |
| **L-549** | *Enterococcus casseliflavus* | Firmicutes, Bacilli, Lactobacillales, Enterococcaceae | 6 | **+** | **+** | **+** | **-** | **+** | **-** | **+** | **+** |
| **L-716** | *Enterococcus casseliflavus* | Firmicutes, Bacilli, Lactobacillales, Enterococcaceae | 6 | **+** | **+** | **+** | **-** | **+** | **-** | **-** | **+** |
| **L-728** | *Enterococcus casseliflavus* | Firmicutes, Bacilli, Lactobacillales, Enterococcaceae | 6 | **+** | **+** | **+** | **-** | **+** | **-** | **-** | **+** |
| **L-880** | *Enterococcus faecium* | Firmicutes, Bacilli, Lactobacillales, Enterococcaceae | 20 | **+** | **+** | **+** | **-** | **-** | **-** | **+** | **+** |
| **L-720** | *Enterococcus faecium* | Firmicutes, Bacilli, Lactobacillales, Enterococcaceae | 22 | **+** | **+** | **+** | **+** | **-** | **-** | **+** | **+** |
| **L-879** | *Enterococcus faecium* | Firmicutes, Bacilli, Lactobacillales, Enterococcaceae | 22 | **+** | **+** | **+** | **-** | **-** | **-** | **+** | **+** |
| **L-883** | *Enterococcus faecium* | Firmicutes, Bacilli, Lactobacillales, Enterococcaceae | 22 | **+** | **+** | **+** | **+** | **+** | **-** | **+** | **+** |
| **L-884** | *Enterococcus faecium* | Firmicutes, Bacilli, Lactobacillales, Enterococcaceae | 22 | **+** | **+** | **+** | **+** | **+** | **-** | **+** | **+** |
| **L-1100** | *Enterococcus faecium* | Firmicutes, Bacilli, Lactobacillales, Enterococcaceae | 22 | **+** | **+** | **+** | **+** | **+** | **-** | **+** | **+** |
| **L-891** | *Enterococcus faecium* | Firmicutes, Bacilli, Lactobacillales, Enterococcaceae | 22 | **+** | **+** | **+** | **+** | **+** | **-** | **+** | **+** |
| **L-1099** | *Enterococcus faecium* | Firmicutes, Bacilli, Lactobacillales, Enterococcaceae | 22 | **+** | **+** | **+** | **+** | **-** | **-** | **+** | **+** |
| **L-990** | *Escherichia hermannii* | Proteobacteria, Gammaproteobacteria, Enterobacteriales, Enterobacteriaceae | 13 | **-** | **-** | **-** | **-** | **-** | **-** | **-** | **+** |
| **L-595** | *Escherichia/ Shigella* sp*.* | Proteobacteria, Gammaproteobacteria, Enterobacteriales, Enterobacteriaceae | 1 | **-** | **-** | **-** | **-** | **-** | **-** | **-** | **+** |
| **L-429** | *Escherichia/ Shigella* sp. | Proteobacteria, Gammaproteobacteria, Enterobacteriales, Enterobacteriaceae | 1 | **-** | **-** | **-** | **-** | **-** | **-** | **-** | **+** |
| **L-1071** | *Escherichia/ Shigella* sp. | Proteobacteria, Gammaproteobacteria, Enterobacteriales, Enterobacteriaceae | 1 | **-** | **-** | **-** | **-** | **-** | **-** | **-** | **+** |
| **L-431** | *Escherichia/ Shigella* sp. | Proteobacteria, Gammaproteobacteria, Enterobacteriales, Enterobacteriaceae | 10 | **-** | **-** | **-** | **-** | **-** | **-** | **-** | **+** |
| **L-982** | *Escherichia/ Shigella* sp. | Proteobacteria, Gammaproteobacteria, Enterobacteriales, Enterobacteriaceae | 10 | **-** | **-** | **-** | **-** | **-** | **-** | **-** | **+** |
| **L-983** | *Escherichia/ Shigella* sp. | Proteobacteria, Gammaproteobacteria, Enterobacteriales, Enterobacteriaceae | 10 | **-** | **-** | **-** | **-** | **-** | **-** | **-** | **+** |
| **L-594** | *Escherichia/ Shigella* sp. | Proteobacteria, Gammaproteobacteria, Enterobacteriales, Enterobacteriaceae | 10 | **-** | **-** | **-** | **-** | **-** | **-** | **-** | **+** |
| **L-433** | *Escherichia/ Shigella* sp. | Proteobacteria, Gammaproteobacteria, Enterobacteriales, Enterobacteriaceae | 10 | **-** | **-** | **-** | **-** | **-** | **-** | **+** | **+** |
| **L-736** | *Escherichia/ Shigella* sp. | Proteobacteria, Gammaproteobacteria, Enterobacteriales, Enterobacteriaceae | 10 | **-** | **-** | **-** | **-** | **-** | **-** | **-** | **+** |
| **L-589** | *Escherichia/ Shigella* sp. | Proteobacteria, Gammaproteobacteria, Enterobacteriales, Enterobacteriaceae | 13 | **-** | **-** | **-** | **-** | **-** | **-** | **-** | **+** |
| **L-590** | *Escherichia/ Shigella* sp. | Proteobacteria, Gammaproteobacteria, Enterobacteriales, Enterobacteriaceae | 13 | **-** | **-** | **-** | **-** | **-** | **-** | **-** | **+** |
| **L-592** | *Escherichia/ Shigella* sp. | Proteobacteria, Gammaproteobacteria, Enterobacteriales, Enterobacteriaceae | 13 | **-** | **-** | **-** | **-** | **-** | **-** | **-** | **+** |
| **L-434** | *Escherichia/ Shigella* sp. | Proteobacteria, Gammaproteobacteria, Enterobacteriales, Enterobacteriaceae | 13 | **-** | **-** | **-** | **-** | **-** | **-** | **-** | **+** |
| **L-745** | *Escherichia/ Shigella* sp. | Proteobacteria, Gammaproteobacteria, Enterobacteriales, Enterobacteriaceae | 13 | **-** | **-** | **-** | **-** | **-** | **-** | **-** | **+** |
| **L-748** | *Escherichia/ Shigella* sp. | Proteobacteria, Gammaproteobacteria, Enterobacteriales, Enterobacteriaceae | 13 | **-** | **-** | **-** | **-** | **-** | **-** | **-** | **+** |
| **L-747** | *Escherichia/ Shigella* sp. | Proteobacteria, Gammaproteobacteria, Enterobacteriales, Enterobacteriaceae | 13 | **-** | **-** | **-** | **-** | **-** | **-** | **-** | **+** |
| **L-436** | *Escherichia/ Shigella* sp. | Proteobacteria, Gammaproteobacteria, Enterobacteriales, Enterobacteriaceae | 22 | **-** | **-** | **-** | **-** | **-** | **-** | **-** | **+** |
| **L-808** | *Escherichia/ Shigella* sp. | Proteobacteria, Gammaproteobacteria, Enterobacteriales, Enterobacteriaceae | 25 | **-** | **-** | **-** | **-** | **-** | **-** | **-** | **+** |
| **L-821** | *Escherichia/ Shigella* sp*.* | Proteobacteria, Gammaproteobacteria, Enterobacteriales, Enterobacteriaceae | 25 | **-** | **-** | **-** | **-** | **-** | **-** | **-** | **+** |
| **L-591** | *Escherichia/ Shigella* sp. | Proteobacteria, Gammaproteobacteria, Enterobacteriales, Enterobacteriaceae | 25 | **-** | **-** | **-** | **-** | **-** | **-** | **-** | **+** |
| **L-437** | *Escherichia/ Shigella* sp. | Proteobacteria, Gammaproteobacteria, Enterobacteriales, Enterobacteriaceae | 25 | **-** | **-** | **-** | **-** | **-** | **-** | **-** | **+** |
| **L-778** | *Escherichia/ Shigella* sp. | Proteobacteria, Gammaproteobacteria, Enterobacteriales, Enterobacteriaceae | 25 | **-** | **-** | **-** | **-** | **-** | **-** | **-** | **+** |
| **L-781** | *Escherichia/ Shigella* sp. | Proteobacteria, Gammaproteobacteria, Enterobacteriales, Enterobacteriaceae | 25 | **-** | **-** | **-** | **-** | **-** | **-** | **-** | **+** |
| **L-785** | *Escherichia/ Shigella* sp. | Proteobacteria, Gammaproteobacteria, Enterobacteriales, Enterobacteriaceae | 25 | **-** | **-** | **-** | **-** | **-** | **-** | **-** | **+** |
| **L-786** | *Escherichia/ Shigella* sp. | Proteobacteria, Gammaproteobacteria, Enterobacteriales, Enterobacteriaceae | 25 | **-** | **-** | **-** | **-** | **-** | **-** | **-** | **+** |
| **L-815** | *Escherichia/ Shigella* sp*.* | Proteobacteria, Gammaproteobacteria, Enterobacteriales, Enterobacteriaceae | 25 | **-** | **-** | **-** | **-** | **-** | **-** | **-** | **+** |
| **L-836** | *Escherichia/ Shigella* sp. | Proteobacteria, Gammaproteobacteria, Enterobacteriales, Enterobacteriaceae | 25 | **-** | **-** | **-** | **-** | **-** | **-** | **-** | **+** |
| **L-940** | *Escherichia/ Shigella* sp. | Proteobacteria, Gammaproteobacteria, Enterobacteriales, Enterobacteriaceae | 25 | **-** | **-** | **-** | **-** | **-** | **-** | **-** | **+** |
| **L-439** | *Escherichia/ Shigella* sp. | Proteobacteria, Gammaproteobacteria, Enterobacteriales, Enterobacteriaceae | 25 | **-** | **-** | **-** | **-** | **-** | **-** | **+** | **+** |
| **L-814** | *Escherichia/ Shigella* sp. | Proteobacteria, Gammaproteobacteria, Enterobacteriales, Enterobacteriaceae | 25 | **-** | **-** | **-** | **-** | **-** | **-** | **-** | **+** |
| **L-593** | *Escherichia/ Shigella* sp. | Proteobacteria, Gammaproteobacteria, Enterobacteriales, Enterobacteriaceae | 25 | **-** | **-** | **-** | **-** | **-** | **-** | **-** | **+** |
| **L-687** | *Escherichia/ Shigella* sp. | Proteobacteria, Gammaproteobacteria, Enterobacteriales, Enterobacteriaceae | 25 | **-** | **-** | **-** | **-** | **-** | **-** | **-** | **+** |
| **L-790** | *Escherichia/ Shigella* sp. | Proteobacteria, Gammaproteobacteria, Enterobacteriales, Enterobacteriaceae | 6 | **-** | **-** | **-** | **-** | **-** | **-** | **-** | **+** |
| **L-396** | *Escherichia/ Shigella* sp. | Proteobacteria, Gammaproteobacteria, Enterobacteriales, Enterobacteriaceae | 6 | **-** | **-** | **-** | **-** | **-** | **-** | **-** | **+** |
| **L-1101** | *Escherichia/ Shigella* sp. | Proteobacteria, Gammaproteobacteria, Enterobacteriales, Enterobacteriaceae | 6 | **-** | **-** | **-** | **-** | **-** | **-** | **-** | **+** |
| **L-553** | *Exiguobacterium* sp. | Firmicutes, Bacilli, Bacillales, Bacillales-Incertae Sedis XII | 10 | **-** | **-** | **-** | **-** | **-** | **-** | **-** | **+** |
| **L-554** | *Exiguobacterium* sp. | Firmicutes, Bacilli, Bacillales, Bacillales-Incertae Sedis XII | 10 | **-** | **-** | **-** | **-** | **-** | **-** | **-** | **+** |
| **L-811** | *Exiguobacterium* sp. | Firmicutes, Bacilli, Bacillales, Bacillales-Incertae Sedis XII | 10 | **-** | **-** | **-** | **-** | **-** | **-** | **-** | **+** |
| **L-875** | *Exiguobacterium* sp. | Firmicutes, Bacilli, Bacillales, Bacillales-Incertae Sedis XII | 10 | **-** | **-** | **-** | **-** | **-** | **-** | **-** | **+** |
| **L-876** | *Exiguobacterium* sp. | Firmicutes, Bacilli, Bacillales, Bacillales-Incertae Sedis XII | 10 | **-** | **-** | **-** | **-** | **-** | **-** | **-** | **+** |
| **L-877** | *Exiguobacterium* sp. | Firmicutes, Bacilli, Bacillales, Bacillales-Incertae Sedis XII | 10 | **-** | **-** | **-** | **-** | **-** | **-** | **-** | **+** |
| **L-878** | *Exiguobacterium* sp. | Firmicutes, Bacilli, Bacillales, Bacillales-Incertae Sedis XII | 10 | **-** | **-** | **-** | **-** | **-** | **-** | **-** | **+** |
| **L-894** | *Exiguobacterium* sp. | Firmicutes, Bacilli, Bacillales, Bacillales-Incertae Sedis XII | 10 | **-** | **-** | **-** | **-** | **-** | **-** | **-** | **+** |
| **L-895** | *Exiguobacterium* sp. | Firmicutes, Bacilli, Bacillales, Bacillales-Incertae Sedis XII | 10 | **-** | **-** | **-** | **-** | **-** | **-** | **-** | **+** |
| **L-896** | *Exiguobacterium* sp. | Firmicutes, Bacilli, Bacillales, Bacillales-Incertae Sedis XII | 10 | **-** | **-** | **-** | **-** | **-** | **-** | **-** | **+** |
| **L-897** | *Exiguobacterium* sp. | Firmicutes, Bacilli, Bacillales, Bacillales-Incertae Sedis XII | 10 | **-** | **-** | **-** | **-** | **-** | **-** | **-** | **+** |
| **L-810** | *Exiguobacterium* sp. | Firmicutes, Bacilli, Bacillales, Bacillales-Incertae Sedis XII | 10 | **-** | **-** | **-** | **-** | **-** | **-** | **-** | **+** |
| **L-1030** | *Exiguobacterium* sp. | Firmicutes, Bacilli, Bacillales, Bacillales-Incertae Sedis XII | 12 | **-** | **-** | **-** | **-** | **-** | **-** | **-** | **+** |
| **L-721** | *Exiguobacterium* sp. | Firmicutes, Bacilli, Bacillales, Bacillales-Incertae Sedis XII | 22 | **+** | **+** | **+** | **+** | **-** | **-** | **+** | **+** |
| **L-732** | *Exiguobacterium* sp. | Firmicutes, Bacilli, Bacillales, Bacillales-Incertae Sedis XII | 23 | **-** | **-** | **-** | **-** | **-** | **-** | **-** | **+** |
| **L-733** | *Exiguobacterium* sp. | Firmicutes, Bacilli, Bacillales, Bacillales-Incertae Sedis XII | 23 | **-** | **-** | **-** | **-** | **-** | **-** | **-** | **+** |
| **L-734** | *Exiguobacterium* sp. | Firmicutes, Bacilli, Bacillales, Bacillales-Incertae Sedis XII | 23 | **-** | **-** | **-** | **-** | **-** | **-** | **-** | **+** |
| **L-933** | *Exiguobacterium* sp. | Firmicutes, Bacilli, Bacillales, Bacillales-Incertae Sedis XII | 23 | **-** | **-** | **-** | **-** | **-** | **-** | **-** | **+** |
| **L-934** | *Exiguobacterium* sp. | Firmicutes, Bacilli, Bacillales, Bacillales-Incertae Sedis XII | 23 | **-** | **-** | **-** | **-** | **-** | **-** | **-** | **+** |
| **L-742** | *Exiguobacterium* sp. | Firmicutes, Bacilli, Bacillales, Bacillales-Incertae Sedis XII | 23 | **-** | **-** | **-** | **-** | **-** | **-** | **-** | **+** |
| **L-952** | *Exiguobacterium* sp*.* | Firmicutes, Bacilli, Bacillales, Bacillales-Incertae Sedis XII | 23 | **-** | **-** | **-** | **-** | **-** | **-** | **-** | **+** |
| **L-440** | *Exiguobacterium* sp. | Firmicutes, Bacilli, Bacillales, Bacillales-Incertae Sedis XII | 25 | **-** | **-** | **-** | **-** | **+** | **-** | **-** | **+** |
| **L-552** | *Exiguobacterium* sp. | Firmicutes, Bacilli, Bacillales, Bacillales-Incertae Sedis XII | 25 | **-** | **-** | **-** | **-** | **+** | **-** | **-** | **+** |
| **L-782** | *Exiguobacterium* sp. | Firmicutes, Bacilli, Bacillales, Bacillales-Incertae Sedis XII | 25 | **-** | **-** | **-** | **-** | **-** | **-** | **-** | **+** |
| **L-783** | *Exiguobacterium* sp. | Firmicutes, Bacilli, Bacillales, Bacillales-Incertae Sedis XII | 25 | **-** | **-** | **-** | **-** | **-** | **-** | **-** | **+** |
| **L-784** | *Exiguobacterium* sp. | Firmicutes, Bacilli, Bacillales, Bacillales-Incertae Sedis XII | 25 | **-** | **-** | **-** | **-** | **-** | **-** | **-** | **+** |
| **L-889** | *Exiguobacterium* sp. | Firmicutes, Bacilli, Bacillales, Bacillales-Incertae Sedis XII | 25 | **-** | **-** | **-** | **-** | **+** | **-** | **-** | **+** |
| **L-898** | *Exiguobacterium* sp. | Firmicutes, Bacilli, Bacillales, Bacillales-Incertae Sedis XII | 25 | **-** | **-** | **-** | **-** | **-** | **-** | **-** | **+** |
| **L-1065** | *Exiguobacterium* sp. | Firmicutes, Bacilli, Bacillales, Bacillales-Incertae Sedis XII | 25 | **-** | **-** | **-** | **-** | **-** | **-** | **-** | **+** |
| **L-1066** | *Exiguobacterium* sp. | Firmicutes, Bacilli, Bacillales, Bacillales-Incertae Sedis XII | 25 | **-** | **-** | **-** | **-** | **-** | **-** | **-** | **+** |
| **L-835** | *Exiguobacterium* sp. | Firmicutes, Bacilli, Bacillales, Bacillales-Incertae Sedis XII | 25 | **-** | **-** | **-** | **-** | **-** | **-** | **-** | **+** |
| **L-822** | *Exiguobacterium* sp. | Firmicutes, Bacilli, Bacillales, Bacillales-Incertae Sedis XII | 25 | **-** | **-** | **-** | **-** | **-** | **-** | **-** | **+** |
| **L-843** | *Exiguobacterium* sp*.* | Firmicutes, Bacilli, Bacillales, Bacillales-Incertae Sedis XII | 29 | **-** | **-** | **-** | **-** | **-** | **-** | **-** | **+** |
| **L-928** | *Exiguobacterium* sp. | Firmicutes, Bacilli, Bacillales, Bacillales-Incertae Sedis XII | 29 | **-** | **+** | **-** | **-** | **-** | **-** | **-** | **+** |
| **L-930** | *Exiguobacterium* sp. | Firmicutes, Bacilli, Bacillales, Bacillales-Incertae Sedis XII | 29 | **-** | **-** | **-** | **-** | **-** | **-** | **-** | **+** |
| **L-931** | *Exiguobacterium* sp. | Firmicutes, Bacilli, Bacillales, Bacillales-Incertae Sedis XII | 29 | **-** | **-** | **-** | **-** | **-** | **-** | **-** | **+** |
| **L-1005** | *Exiguobacterium* sp. | Firmicutes, Bacilli, Bacillales, Bacillales-Incertae Sedis XII | 29 | **-** | **-** | **-** | **-** | **-** | **-** | **-** | **+** |
| **L-1006** | *Exiguobacterium* sp. | Firmicutes, Bacilli, Bacillales, Bacillales-Incertae Sedis XII | 29 | **-** | **-** | **-** | **-** | **-** | **-** | **-** | **+** |
| **L-1008** | *Exiguobacterium* sp. | Firmicutes, Bacilli, Bacillales, Bacillales-Incertae Sedis XII | 29 | **-** | **-** | **-** | **-** | **-** | **-** | **-** | **+** |
| **L-1048** | *Exiguobacterium* sp. | Firmicutes, Bacilli, Bacillales, Bacillales-Incertae Sedis XII | 29 | **-** | **-** | **-** | **-** | **-** | **-** | **-** | **+** |
| **L-646** | *Exiguobacterium* sp. | Firmicutes, Bacilli, Bacillales, Bacillales-Incertae Sedis XII | 34 | **-** | **-** | **-** | **-** | **-** | **-** | **-** | **+** |
| **L-804** | *Exiguobacterium* sp. | Firmicutes, Bacilli, Bacillales, Bacillales-Incertae Sedis XII | 34 | **-** | **-** | **-** | **-** | **-** | **-** | **-** | **+** |
| **L-805** | *Exiguobacterium* sp. | Firmicutes, Bacilli, Bacillales, Bacillales-Incertae Sedis XII | 34 | **-** | **-** | **-** | **-** | **-** | **-** | **-** | **+** |
| **L-1089** | *Exiguobacterium* sp. | Firmicutes, Bacilli, Bacillales, Bacillales-Incertae Sedis XII | 36 | **-** | **-** | **-** | **-** | **-** | **-** | **-** | **+** |
| **L-1085** | *Exiguobacterium* sp. | Firmicutes, Bacilli, Bacillales, Bacillales-Incertae Sedis XII | 36 | **-** | **-** | **-** | **-** | **-** | **-** | **-** | **+** |
| **L-1090** | *Exiguobacterium* sp. | Firmicutes, Bacilli, Bacillales, Bacillales-Incertae Sedis XII | 36 | **-** | **-** | **-** | **-** | **-** | **-** | **-** | **+** |
| **L-384** | *Exiguobacterium* sp. | Firmicutes, Bacilli, Bacillales, Bacillales-Incertae Sedis XII | 4 | **-** | **-** | **-** | **-** | **-** | **-** | **-** | **+** |
| **L-936** | *Flavobacterium lindanitolerans* | Bacteroidetes, Flavobacteria, Flavobacteriales, Flavobacteriaceae | 25 | **-** | **-** | **-** | **-** | **-** | **-** | **-** | **+** |
| **L-939** | *Flavobacterium lindanitolerans* | Bacteroidetes, Flavobacteria, Flavobacteriales, Flavobacteriaceae | 25 | **-** | **-** | **-** | **-** | **-** | **-** | **-** | **+** |
| **L-948** | *Gordonia paraffinivorans* | Actinobacteria, Actinobacteria, Actinomycetales, Nocardiaceae | 6 | **-** | **-** | **-** | **-** | **-** | **-** | **+** | **+** |
| **L-953** | *Gordonia paraffinivorans* | Actinobacteria, Actinobacteria, Actinomycetales, Nocardiaceae | 25 | **-** | **-** | **-** | **-** | **-** | **-** | **-** | **+** |
| **L-964** | *Gordonia paraffinivorans* | Actinobacteria, Actinobacteria, Actinomycetales, Nocardiaceae | 31 | **-** | **-** | **-** | **-** | **-** | **-** | **-** | **+** |
| **L-965** | *Gordonia paraffinivorans* | Actinobacteria, Actinobacteria, Actinomycetales, Nocardiaceae | 31 | **-** | **-** | **-** | **-** | **-** | **-** | **-** | **+** |
| **L-871** | *Gordonia polyisoprenivorans* | Actinobacteria, Actinobacteria, Actinomycetales, Nocardiaceae | 15 | **-** | **-** | **-** | **-** | **-** | **-** | **-** | **+** |
| **L-988** | *Klebsiella oxytoca* | Proteobacteria, Gammaproteobacteria, Enterobacteriales, Enterobacteriaceae | 1 | **-** | **-** | **-** | **-** | **-** | **-** | **-** | **+** |
| **L-666** | *Klebsiella oxytoca* | Proteobacteria, Gammaproteobacteria, Enterobacteriales, Enterobacteriaceae | 1 | **-** | **-** | **-** | **-** | **-** | **-** | **-** | **+** |
| **L-668** | *Klebsiella oxytoca* | Proteobacteria, Gammaproteobacteria, Enterobacteriales, Enterobacteriaceae | 1 | **-** | **-** | **-** | **-** | **-** | **-** | **-** | **+** |
| **L-671** | *Klebsiella oxytoca* | Proteobacteria, Gammaproteobacteria, Enterobacteriales, Enterobacteriaceae | 1 | **-** | **-** | **-** | **-** | **-** | **-** | **-** | **+** |
| **L-555** | *Klebsiella oxytoca* | Proteobacteria, Gammaproteobacteria, Enterobacteriales, Enterobacteriaceae | 2 | **-** | **-** | **-** | **-** | **-** | **-** | **-** | **+** |
| **L-1013** | *Klebsiella pneumoniae* | Proteobacteria, Gammaproteobacteria, Enterobacteriales, Enterobacteriaceae | 13 | **-** | **-** | **-** | **-** | **-** | **+** | **-** | **+** |
| **L-739** | *Klebsiella pneumoniae* | Proteobacteria, Gammaproteobacteria, Enterobacteriales, Enterobacteriaceae | 13 | **-** | **-** | **-** | **-** | **-** | **-** | **-** | **+** |
| **L-1014** | *Klebsiella pneumoniae* | Proteobacteria, Gammaproteobacteria, Enterobacteriales, Enterobacteriaceae | 2 | **-** | **-** | **-** | **-** | **-** | **-** | **-** | **+** |
| **L-556** | *Klebsiella pneumoniae* | Proteobacteria, Gammaproteobacteria, Enterobacteriales, Enterobacteriaceae | 2 | **-** | **-** | **-** | **-** | **-** | **-** | **-** | **+** |
| **L-1015** | *Klebsiella pneumoniae* | Proteobacteria, Gammaproteobacteria, Enterobacteriales, Enterobacteriaceae | 2 | **-** | **-** | **-** | **-** | **-** | **-** | **-** | **+** |
| **L-714** | *Klebsiella pneumoniae* | Proteobacteria, Gammaproteobacteria, Enterobacteriales, Enterobacteriaceae | 2 | **-** | **-** | **-** | **-** | **-** | **-** | **-** | **+** |
| **L-1043** | *Klebsiella pneumoniae* | Proteobacteria, Gammaproteobacteria, Enterobacteriales, Enterobacteriaceae | 20 | **-** | **-** | **-** | **-** | **-** | **-** | **-** | **+** |
| **L-901** | *Klebsiella pneumoniae* | Proteobacteria, Gammaproteobacteria, Enterobacteriales, Enterobacteriaceae | 20 | **-** | **-** | **-** | **-** | **+** | **-** | **-** | **+** |
| **L-902** | *Klebsiella pneumoniae* | Proteobacteria, Gammaproteobacteria, Enterobacteriales, Enterobacteriaceae | 20 | **-** | **-** | **-** | **-** | **+** | **-** | **-** | **+** |
| **L-903** | *Klebsiella pneumoniae* | Proteobacteria, Gammaproteobacteria, Enterobacteriales, Enterobacteriaceae | 20 | **-** | **-** | **-** | **-** | **+** | **-** | **-** | **+** |
| **L-1044** | *Klebsiella pneumoniae* | Proteobacteria, Gammaproteobacteria, Enterobacteriales, Enterobacteriaceae | 20 | **-** | **-** | **-** | **-** | **+** | **-** | **-** | **+** |
| **L-395** | *Klebsiella pneumoniae* | Proteobacteria, Gammaproteobacteria, Enterobacteriales, Enterobacteriaceae | 6 | **-** | **-** | **-** | **-** | **-** | **-** | **-** | **+** |
| **L-1731** | *Kocuria kristinae* | Actinobacteria, Actinobacteria, Actinomycetales, Micrococcineae | 2 |  |  |  |  |  |  |  |  |
| **L-644** | *Kocuria rhizophila* | Actinobacteria, Actinobacteria, Actinomycetales, Micrococcineae | 33 | **-** | **-** | **-** | **-** | **+** | **-** | **-** | **+** |
| **L-917** | *Kurthia gibsonii* | Firmicutes, Bacilli, Bacillales, Planococcaceae | 12 | **+** | **+** | **+** | **-** | **+** | **-** | **-** | **+** |
| **L-919** | *Kurthia gibsonii* | Firmicutes, Bacilli, Bacillales, Planococcaceae | 12 | **+** | **+** | **+** | **-** | **+** | **-** | **-** | **+** |
| **L-920** | *Kurthia gibsonii* | Firmicutes, Bacilli, Bacillales, Planococcaceae | 12 | **+** | **+** | **+** | **-** | **+** | **-** | **-** | **+** |
| **L-921** | *Kurthia gibsonii* | Firmicutes, Bacilli, Bacillales, Planococcaceae | 12 | **+** | **+** | **+** | **-** | **-** | **-** | **-** | **+** |
| **L-1031** | *Kurthia gibsonii* | Firmicutes, Bacilli, Bacillales, Planococcaceae | 12 | **+** | **+** | **+** | **-** | **+** | **-** | **-** | **+** |
| **L-557** | *Kurthia gibsonii* | Firmicutes, Bacilli, Bacillales,Planococcaceae | 22 | **+** | **+** | **+** | **-** | **+** | **-** | **-** | **+** |
| **L-392** | *Kurthia gibsonii* | Firmicutes, Bacilli, Bacillales,Planococcaceae | 6 | **+** | **+** | **+** | **-** | **+** | **+** | **-** | **+** |
| **L-398** | *Lysinibacillus fusiformis* | Firmicutes, Bacilli, Bacillales,Planococcaceae | 10 | **-** | **+** | **+** | **-** | **+** | **-** | **-** | **+** |
| **L-1026** | *Lysinibacillus fusiformis* | Firmicutes, Bacilli, Bacillales,Planococcaceae | 20 | **-** | **+** | **+** | **-** | **+** | **-** | **-** | **+** |
| **L-855** | *Lysinibacillus fusiformis* | Firmicutes, Bacilli, Bacillales, Planococcaceae | 30 | **+** | **+** | **+** | **-** | **+** | **-** | **-** | **+** |
| **L-1045** | *Lysinibacillus fusiformis* | Firmicutes, Bacilli, Bacillales,Planococcaceae | 31 | **-** | **+** | **+** | **-** | **+** | **-** | **-** | **+** |
| **L-967** | *Lysinibacillus fusiformis* | Firmicutes, Bacilli, Bacillales, Planococcaceae | 32 | **-** | **+** | **+** | **-** | **+** | **-** | **-** | **+** |
| **L-1041** | *Lysinibacillus fusiformis* | Firmicutes, Bacilli, Bacillales,Planococcaceae | 32 | **+** | **+** | **+** | **-** | **+** | **-** | **-** | **+** |
| **L-558** | *Lysinibacillus fusiformis* | Firmicutes, Bacilli, Bacillales,Planococcaceae | 5 | **-** | **+** | **+** | **-** | **+** | **-** | **-** | **+** |
| **L-652** | *Microbacterium aurum* | Actinobacteria, Actinobacteria, Actinomycetales, Micrococcineae | 31 | **-** | **+** | **+** | **+** | **+** | **-** | **+** | **+** |
| **L-559** | *Microbacterium lacticum* | Actinobacteria, Actinobacteria, Actinomycetales, Microbacteriaceae | 7 | **-** | **+** | **+** | **-** | **+** | **-** | **-** | **+** |
| **L-560** | *Microbacterium lacticum* | Actinobacteria, Actinobacteria, Actinomycetales, Microbacteriaceae | 7 | **-** | **+** | **+** | **-** | **+** | **-** | **-** | **+** |
| **L-596** | *Microbacterium oxydans* | Actinobacteria, Actinobacteria, Actinomycetales, Microbacteriaceae | 5 | **-** | **+** | **+** | **+** | **+** | **-** | **-** | **+** |
| **L-962** | *Microbacterium oxydans* | Actinobacteria, Actinobacteria, Actinomycetales, Microbacteriaceae | 13 | **+** | **-** | **+** | **-** | **-** | **-** | **-** | **+** |
| **L-430** | *Microbacterium* sp. | Actinobacteria, Actinobacteria, Actinomycetales, Microbacteriaceae | 10 | **+** | **+** | **+** | **-** | **+** | **-** | **+** | **+** |
| **L-735** | *Micrococcus luteus* | Actinobacteria, Actinobacteria, Actinomycetales, Micrococcineae | 10 | **-** | **-** | **-** | **-** | **+** | **-** | **-** | **+** |
| **L-768** | *Micrococcus luteus* | Actinobacteria, Actinobacteria, Actinomycetales, Micrococcaceae | 11 | **-** | **+** | **-** | **-** | **+** | **-** | **-** | **+** |
| **L-769** | *Micrococcus luteus* | Actinobacteria, Actinobacteria, Actinomycetales, Micrococcaceae | 11 | **-** | **+** | **-** | **-** | **-** | **-** | **-** | **+** |
| **L-772** | *Micrococcus luteus* | Actinobacteria, Actinobacteria, Actinomycetales, Micrococcaceae | 11 | **-** | **+** | **-** | **-** | **+** | **-** | **-** | **+** |
| **L-774** | *Micrococcus luteus* | Actinobacteria, Actinobacteria, Actinomycetales, Micrococcaceae | 11 | **-** | **+** | **-** | **-** | **-** | **-** | **-** | **+** |
| **L-673** | *Micrococcus luteus* | Actinobacteria, Actinobacteria, Actinomycetales, Micrococcineae | 20 | **-** | **+** | **-** | **-** | **+** | **-** | **-** | **+** |
| **L-731** | *Micrococcus luteus* | Actinobacteria, Actinobacteria, Actinomycetales, Micrococcineae | 23 | **-** | **+** | **-** | **-** | **+** | **-** | **-** | **+** |
| **L-743** | *Micrococcus luteus* | Actinobacteria, Actinobacteria, Actinomycetales, Micrococcineae | 23 | **-** | **-** | **-** | **-** | **+** | **-** | **-** | **+** |
| **L-744** | *Micrococcus luteus* | Actinobacteria, Actinobacteria, Actinomycetales, Micrococcineae | 23 | **-** | **-** | **-** | **-** | **+** | **-** | **-** | **+** |
| **L-1003** | *Micrococcus luteus* | Actinobacteria, Actinobacteria, Actinomycetales, Micrococcaceae | 29 | **-** | **+** | **-** | **-** | **+** | **-** | **-** | **+** |
| **L-1004** | *Micrococcus luteus* | Actinobacteria, Actinobacteria, Actinomycetales, Micrococcaceae | 29 | **-** | **+** | **-** | **-** | **+** | **-** | **-** | **+** |
| **L-926** | *Micrococcus luteus* | Actinobacteria, Actinobacteria, Actinomycetales, Micrococcaceae | 30 | **-** | **-** | **-** | **-** | **+** | **-** | **-** | **+** |
| **L-927** | *Micrococcus luteus* | Actinobacteria, Actinobacteria, Actinomycetales, Micrococcaceae | 30 | **-** | **-** | **-** | **-** | **+** | **-** | **-** | **+** |
| **L-654** | *Micrococcus luteus* | Actinobacteria, Actinobacteria, Actinomycetales, Micrococcineae | 31 | **-** | **+** | **-** | **-** | **+** | **-** | **-** | **+** |
| **L-1046** | *Micrococcus luteus* | Actinobacteria, Actinobacteria, Actinomycetales, Micrococcaceae | 31 | **-** | **+** | **-** | **-** | **+** | **-** | **-** | **+** |
| **L-1050** | *Micrococcus luteus* | Actinobacteria, Actinobacteria, Actinomycetales, Micrococcaceae | 31 | **-** | **+** | **-** | **-** | **+** | **-** | **-** | **+** |
| **L-1051** | *Micrococcus luteus* | Actinobacteria, Actinobacteria, Actinomycetales, Micrococcaceae | 31 | **-** | **+** | **-** | **-** | **+** | **-** | **-** | **+** |
| **L-653** | *Micrococcus luteus* | Actinobacteria, Actinobacteria, Actinomycetales, Micrococcineae | 31 | **-** | **-** | **-** | **-** | **+** | **-** | **-** | **+** |
| **L-650** | *Micrococcus luteus* | Actinobacteria, Actinobacteria, Actinomycetales, Micrococcineae | 34 | **-** | **-** | **-** | **-** | **+** | **-** | **-** | **+** |
| **L-1086** | *Micrococcus luteus* | Actinobacteria, Actinobacteria, Actinomycetales, Micrococcaceae | 36 | **-** | **+** | **-** | **-** | **-** | **-** | **-** | **+** |
| **L-1087** | *Micrococcus luteus* | Actinobacteria, Actinobacteria, Actinomycetales, Micrococcaceae | 36 | **-** | **-** | **-** | **-** | **-** | **-** | **-** | **+** |
| **L-1088** | *Micrococcus luteus* | Actinobacteria, Actinobacteria, Actinomycetales, Micrococcaceae | 36 | **-** | **+** | **-** | **-** | **+** | **-** | **-** | **+** |
| **L-561** | *Micrococcus luteus* | Actinobacteria, Actinobacteria, Actinomycetales, Micrococcaceae | 7 | **-** | **-** | **-** | **-** | **+** | **-** | **-** | **+** |
| **L-956** | *Micrococcus* sp. | Actinobacteria, Actinobacteria, Actinomycetales, Micrococcaceae | 15 | **-** | **-** | **-** | **-** | **+** | **-** | **-** | **+** |
| **L-741** | *Micrococcus* sp. | Actinobacteria, Actinobacteria, Actinomycetales, Micrococcineae | 23 | **-** | **-** | **-** | **-** | **+** | **-** | **-** | **+** |
| **L-562** | *Micrococcus* sp. | Actinobacteria, Actinobacteria, Actinomycetales, Micrococcaceae | 30 | **-** | **-** | **-** | **-** | **+** | **-** | **-** | **+** |
| **L-651** | *Micrococcus* sp. | Actinobacteria, Actinobacteria, Actinomycetales, Micrococcineae | 34 | **-** | **-** | **-** | **-** | **+** | **-** | **-** | **+** |
| **L-648** | *Micrococcus* sp. | Actinobacteria, Actinobacteria, Actinomycetales, Micrococcineae | 34 | **-** | **+** | **-** | **-** | **+** | **-** | **-** | **+** |
| **L-730** | *Micrococcus* sp. | Actinobacteria, Actinobacteria, Actinomycetales, Micrococcineae | 6 | **-** | **-** | **-** | **-** | **+** | **-** | **-** | **+** |
| **L-563** | *Micrococcus* sp. | Actinobacteria, Actinobacteria, Actinomycetales, Micrococcaceae | 7 | **-** | **+** | **-** | **-** | **+** | **-** | **-** | **+** |
| **L-1024** | *Naumannella halotolerans* | Actinobacteria, Actinobacteria, Actinomycetales, Propionibacteriaceae | 14 | **-** | **-** | **-** | **-** | **+** | **-** | **-** | **+** |
| **L-979** | *Ochrobactrum* sp. | Proteobacteria, Alphaproteobacteria, Rhizobiales, Brucellaceae | 10 | **+** | **+** | **-** | **+** | **-** | **-** | **+** | **+** |
| **L-960** | *Ochrobactrum* sp. | Proteobacteria, Alphaproteobacteria, Rhizobiales, Brucellaceae | 2 | **+** | **+** | **+** | **+** | **+** | **-** | **+** | **+** |
| **L-947** | *Paenibacillus* sp. | Firmicutes, Bacilli, Bacillales, Paenibacillaceae 1 | 20 | **+** | **+** | **-** | **-** | **-** | **-** | **-** | **+** |
| **L-564** | *Paenibacillus* sp. | Firmicutes, Bacilli, Bacillales, Paenibacillaceae 1 | 27 | **-** | **+** | **-** | **-** | **+** | **-** | **-** | **+** |
| **L-565** | *Paenibacillus* sp. | Firmicutes, Bacilli, Bacillales, Paenibacillaceae 1 | 27 | **-** | **+** | **-** | **-** | **+** | **-** | **-** | **+** |
| **L-1002** | *Paenibacillus* sp. | Firmicutes, Bacilli, Bacillales, Paenibacillaceae 1 | 27 | **-** | **+** | **-** | **-** | **-** | **-** | **-** | **+** |
| **L-1001** | *Paenibacillus* sp. | Firmicutes, Bacilli, Bacillales, Paenibacillaceae 1 | 27 | **-** | **-** | **-** | **-** | **-** | **-** | **-** | **+** |
| **L-1103** | *Paenibacillus* sp. | Firmicutes, Bacilli, Bacillales, Paenibacillaceae 1 | 27 | **-** | **+** | **+** | **+** | **-** | **-** | **-** | **+** |
| **L-796** | *Paenibacillus* sp. | Firmicutes, Bacilli, Bacillales, Paenibacillaceae 1 | 27 | **-** | **+** | **+** | **+** | **-** | **-** | **-** | **+** |
| **L-906** | *Paenibacillus* sp. | Firmicutes, Bacilli, Bacillales, Paenibacillaceae 1 | 28 | **-** | **+** | **+** | **+** | **-** | **-** | **-** | **+** |
| **L-567** | *Paenibacillus* sp. | Firmicutes, Bacilli, Bacillales, Paenibacillaceae 1 | 3 | **-** | **+** | **-** | **-** | **+** | **-** | **+** | **+** |
| **L-568** | *Paenibacillus* sp. | Firmicutes, Bacilli, Bacillales, Paenibacillaceae 1 | 3 | **-** | **+** | **-** | **-** | **+** | **-** | **+** | **+** |
| **L-968** | *Paenibacillus* sp. | Firmicutes, Bacilli, Bacillales, Paenibacillaceae | 32 | **-** | **-** | **-** | **-** | **+** | **-** | **-** | **+** |
| **L-659** | *Paenibacillus* sp. | Firmicutes, Bacilli, Bacillales, Paenibacillaceae 1 | 32 | **-** | **-** | **-** | **+** | **+** | **-** | **-** | **+** |
| **L-980** | *Paenibacillus* sp. | Firmicutes, Bacilli, Bacillales, Paenibacillaceae | 33 | **-** | **+** | **-** | **-** | **-** | **-** | **-** | **+** |
| **L-1074** | *Paenibacillus* sp. | Firmicutes, Bacilli, Bacillales, Paenibacillaceae 1 | 35 | **-** | **-** | **-** | **+** | **-** | **-** | **-** | **+** |
| **L-1075** | *Paenibacillus* sp. | Firmicutes, Bacilli, Bacillales, Paenibacillaceae 1 | 35 | **-** | **-** | **-** | **+** | **-** | **-** | **-** | **+** |
| **L-1022** | *Paenibacillus* sp. | Firmicutes, Bacilli, Bacillales, Paenibacillaceae 1 | 35 | **+** | **-** | **-** | **+** | **-** | **-** | **-** | **+** |
| **L-1076** | *Paenibacillus* sp. | Firmicutes, Bacilli, Bacillales, Paenibacillaceae 1 | 35 | **-** | **-** | **+** | **+** | **-** | **-** | **-** | **+** |
| **L-566** | *Paenibacillus* sp. | Firmicutes, Bacilli, Bacillales, Paenibacillaceae 1 | 4 | **-** | **+** | **-** | **-** | **+** | **-** | **+** | **+** |
| **L-992** | *Paenibacillus* sp. | Firmicutes, Bacilli, Bacillales, Paenibacillaceae | 5 | **-** |  |  |  |  |  | **-** | **+** |
| **L-816** | *Paenibacillus* sp. | Firmicutes, Bacilli, Bacillales, Paenibacillaceae 1 | 9 | **-** | **+** | **+** | **+** | **-** | **-** | **+** | **+** |
| **L-848** | *Paenibacillus* sp. | Firmicutes, Bacilli, Bacillales, Paenibacillaceae 1 | 9 | **-** | **+** | **-** | **-** | **+** | **-** | **+** | **+** |
| **L-746** | *Pantoea agglomerans* | Proteobacteria, Gammaproteobacteria, Enterobacteriales, Enterobacteriaceae | 13 |  |  |  |  |  |  |  |  |
| **L-994** | *Pseudomonas aeruginosa* | Proteobacteria, Gammaproteobacteria, Pseudomonadales, Pseudomonadaceae | 1 | **+** | **+** | **+** | **+** | **+** | **-** | **+** | **+** |
| **L-597** | *Pseudomonas aeruginosa* | Proteobacteria, Gammaproteobacteria, Pseudomonadales, Pseudomonadaceae | 13 | **+** | **-** | **+** | **-** | **-** | **-** | **+** | **+** |
| **L-1061** | *Pseudomonas aeruginosa* | Proteobacteria, Gammaproteobacteria, Pseudomonadales, Pseudomonadaceae | 13 | **+** | **-** | **+** | **+** | **-** | **-** | **+** | **+** |
| **L-996** | *Pseudomonas aeruginosa* | Proteobacteria, Gammaproteobacteria, Pseudomonadales, Pseudomonadaceae | 19 | **+** | **-** | **+** | **+** | **+** | **-** | **+** | **+** |
| **L-997** | *Pseudomonas aeruginosa* | Proteobacteria, Gammaproteobacteria, Pseudomonadales, Pseudomonadaceae | 19 | **+** | **-** | **+** | **+** | **-** | **-** | **+** | **+** |
| **L-998** | *Pseudomonas aeruginosa* | Proteobacteria, Gammaproteobacteria, Pseudomonadales, Pseudomonadaceae | 19 | **+** | **-** | **+** | **+** | **-** | **-** | **+** | **+** |
| **L-999** | *Pseudomonas aeruginosa* | Proteobacteria, Gammaproteobacteria, Pseudomonadales, Pseudomonadaceae | 19 | **+** | **-** | **+** | **+** | **-** | **-** | **+** | **+** |
| **L-685** | *Pseudomonas aeruginosa* | Proteobacteria, Gammaproteobacteria, Pseudomonadales, Pseudomonadaceae | 2 | **+** | **-** | **+** | **+** | **-** | **-** | **+** | **+** |
| **L-686** | *Pseudomonas aeruginosa* | Proteobacteria, Gammaproteobacteria, Pseudomonadales, Pseudomonadaceae | 2 | **+** | **-** | **+** | **+** | **-** | **-** | **+** | **+** |
| **L-993** | *Pseudomonas aeruginosa* | Proteobacteria, Gammaproteobacteria, Pseudomonadales, Pseudomonadaceae | 2 | **+** | **-** | **+** | **+** | **+** | **-** | **+** | **+** |
| **L-995** | *Pseudomonas aeruginosa* | Proteobacteria, Gammaproteobacteria, Pseudomonadales, Pseudomonadaceae | 24 | **+** | **-** | **+** | **+** | **+** | **-** | **+** | **+** |
| **L-912** | *Pseudomonas aeruginosa* | Proteobacteria, Gammaproteobacteria, Pseudomonadales, Pseudomonadaceae | 29 | **+** | **-** | **+** | **+** | **+** | **-** | **+** | **+** |
| **L-1072** | *Pseudomonas pseudoalcaligenes* | Proteobacteria, Gammaproteobacteria, Pseudomonadales, Pseudomonadaceae | 1 | **+** | **-** | **-** | **-** | **-** | **-** | **-** | **+** |
| **L-722** | *Pseudomonas pseudoalcaligenes* | Proteobacteria, Gammaproteobacteria, Pseudomonadales, Pseudomonadaceae | 22 | **-** | **-** | **+** | **-** | **-** | **-** | **-** | **+** |
| **L-881** | *Pseudomonas pseudoalcaligenes* | Proteobacteria, Gammaproteobacteria, Pseudomonadales, Pseudomonadaceae | 22 | **+** | **-** | **+** | **+** | **-** | **-** | **-** | **+** |
| **L-893** | *Pseudomonas pseudoalcaligenes* | Proteobacteria, Gammaproteobacteria, Pseudomonadales, Pseudomonadaceae | 22 | **+** | **-** | **-** | **+** | **-** | **-** | **-** | **+** |
| **L-813** | *Pseudomonas pseudoalcaligenes* | Proteobacteria, Gammaproteobacteria, Pseudomonadales, Pseudomonadaceae | 22 | **-** | **-** | **-** | **-** | **-** | **-** | **-** | **+** |
| **L-890** | *Pseudomonas pseudoalcaligenes* | Proteobacteria, Gammaproteobacteria, Pseudomonadales, Pseudomonadaceae | 22 | **-** | **-** | **-** | **+** | **-** | **-** | **-** | **+** |
| **L-1073** | *Pseudomonas pseudoalcaligenes* | Proteobacteria, Gammaproteobacteria, Pseudomonadales, Pseudomonadaceae | 23 | **-** | **-** | **-** | **-** | **-** | **-** | **-** | **+** |
| **L-1079** | *Pseudomonas pseudoalcaligenes* | Proteobacteria, Gammaproteobacteria, Pseudomonadales, Pseudomonadaceae | 29 | **+** | **-** | **-** | **-** | **-** | **-** | **-** | **+** |
| **L-1080** | *Pseudomonas pseudoalcaligenes* | Proteobacteria, Gammaproteobacteria, Pseudomonadales, Pseudomonadaceae | 29 | **+** | **-** | **-** | **-** | **-** | **-** | **-** | **+** |
| **L-1081** | *Pseudomonas pseudoalcaligenes* | Proteobacteria, Gammaproteobacteria, Pseudomonadales, Pseudomonadaceae | 29 | **+** | **-** | **-** | **-** | **-** | **-** | **-** | **+** |
| **L-1082** | *Pseudomonas pseudoalcaligenes* | Proteobacteria, Gammaproteobacteria, Pseudomonadales, Pseudomonadaceae | 29 | **-** | **-** | **-** | **-** | **-** | **-** | **-** | **+** |
| **L-970** | *Pseudomonas pseudoalcaligenes* | Proteobacteria, Gammaproteobacteria, Pseudomonadales, Pseudomonadaceae | 29 | **-** | **-** | **-** | **-** | **-** | **-** | **-** | **+** |
| **L-1083** | *Pseudomonas pseudoalcaligenes* | Proteobacteria, Gammaproteobacteria, Pseudomonadales, Pseudomonadaceae | 8 | **+** | **+** | **-** | **-** | **-** | **-** | **-** | **+** |
| **L-435** | *Pseudomonas* sp. | Proteobacteria, Gammaproteobacteria, Pseudomonadales, Pseudomonadaceae | 22 | **-** | **-** | **-** | **-** | **-** | **-** | **-** | **+** |
| **L-569** | *Pseudomonas* sp. | Proteobacteria, Gammaproteobacteria, Pseudomonadales, Pseudomonadaceae | 22 | **+** | **-** | **-** | **-** | **-** | **-** | **-** | **+** |
| **L-989** | *Pseudomonas* sp. | Proteobacteria, Gammaproteobacteria, Pseudomonadales, Pseudomonadaceae | 24 | **-** | **-** | **-** | **+** | **-** | **-** | **-** | **+** |
| **L-388** | *Pseudomonas* sp. | Proteobacteria, Gammaproteobacteria, Pseudomonadales, Pseudomonadaceae | 4 | **+** | **-** | **-** | **-** | **-** | **-** | **-** | **+** |
| **L-389** | *Pseudomonas* sp. | Proteobacteria, Gammaproteobacteria, Pseudomonadales, Pseudomonadaceae | 4 | **+** | **-** | **-** | **-** | **-** | **-** | **-** | **+** |
| **L-387** | *Pseudomonas* sp. | Proteobacteria, Gammaproteobacteria, Pseudomonadales, Pseudomonadaceae | 4 | **+** | **-** | **-** | **-** | **-** | **-** | **-** | **+** |
| **L-410** | *Pseudomonas* sp. | Proteobacteria, Gammaproteobacteria, Pseudomonadales, Pseudomonadaceae | 6 | **+** | **-** | **-** | **-** | **-** | **+** | **-** | **+** |
| **L-1058** | *Pseudomonas stutzeri* | Proteobacteria, Gammaproteobacteria, Pseudomonadales, Pseudomonadaceae | 25 | **-** | **-** | **+** | **-** | **-** | **-** | **-** | **+** |
| **L-1084** | *Pseudomonas stutzeri* | Proteobacteria, Gammaproteobacteria, Pseudomonadales, Pseudomonadaceae | 8 | **+** | **-** | **+** | **-** | **-** | **-** | **-** | **+** |
| **L-1107** | *Raoultella ornithinolytica* | Proteobacteria, Gammaproteobacteria, Enterobacteriales, Enterobacteriaceae | 12 | **-** | **-** | **-** | **-** | **-** | **-** | **-** | **+** |
| **L-1057** | *Raoultella ornithinolytica* | Proteobacteria, Gammaproteobacteria, Enterobacteriales, Enterobacteriaceae | 2 | **-** | **-** | **-** | **+** | **-** | **-** | **-** | **+** |
| **L-570** | *Raoultella ornithinolytica* | Proteobacteria, Gammaproteobacteria, Enterobacteriales, Enterobacteriaceae | 2 | **-** | **-** | **-** | **+** | **-** | **-** | **-** | **+** |
| **L-658** | *Raoultella ornithinolytica* | Proteobacteria, Gammaproteobacteria, Enterobacteriales, Enterobacteriaceae | 2 | **-** | **-** | **-** | **+** | **-** | **-** | **-** | **+** |
| **L-677** | *Raoultella ornithinolytica* | Proteobacteria, Gammaproteobacteria, Enterobacteriales, Enterobacteriaceae | 2 | **-** | **-** | **-** | **-** | **-** | **-** | **-** | **+** |
| **L-679** | *Raoultella ornithinolytica* | Proteobacteria, Gammaproteobacteria, Enterobacteriales, Enterobacteriaceae | 2 | **-** | **-** | **-** | **+** | **-** | **-** | **-** | **+** |
| **L-684** | *Raoultella ornithinolytica* | Proteobacteria, Gammaproteobacteria, Enterobacteriales, Enterobacteriaceae | 2 | **-** | **-** | **-** | **+** | **-** | **-** | **-** | **+** |
| **L-701** | *Raoultella ornithinolytica* | Proteobacteria, Gammaproteobacteria, Enterobacteriales, Enterobacteriaceae | 2 | **-** | **-** | **-** | **+** | **-** | **-** | **-** | **+** |
| **L-704** | *Raoultella ornithinolytica* | Proteobacteria, Gammaproteobacteria, Enterobacteriales, Enterobacteriaceae | 2 | **-** | **-** | **-** | **+** | **-** | **-** | **-** | **+** |
| **L-706** | *Raoultella ornithinolytica* | Proteobacteria, Gammaproteobacteria, Enterobacteriales, Enterobacteriaceae | 2 | **-** | **-** | **-** | **+** | **-** | **-** | **-** | **+** |
| **L-750** | *Raoultella ornithinolytica* | Proteobacteria, Gammaproteobacteria, Enterobacteriales, Enterobacteriaceae | 2 | **-** | **-** | **-** | **+** | **-** | **-** | **-** | **+** |
| **L-413** | *Roseomonas cervicalis* | Proteobacteria, Alphaproteobacteria, Rhodospirillales, Acetobacteraceae | 6 | **+** | **+** | **-** | **-** | **-** | **-** | **-** | **+** |
| **L-868** | *Roseomonas cervicalis* | Proteobacteria, Alphaproteobacteria, Rhodospirillales, Acetobacteraceae | 24 | **+** | **+** | **-** | **-** | **-** | **-** | **-** | **+** |
| **L-973** | *Sphingobacterium multivorum* | Bacterioidetes, Sphingobacteria, Sphingobacteriales, Sphingobacteriaceae | 30 | **+** | **+** | **+** | **+** | **+** | **-** | **+** | **+** |
| **L-932** | *Sphingomonas mucosissima* | Proteobacteria, Alphaproteobacteria, Sphingomonadales, Sphingomonadaceae | 24 | **-** | **-** | **-** | **-** | **-** | **-** | **-** | **+** |
| **L-1016** | *Sphingomonas paucimobilis* | Proteobacteria, Alphaproteobacteria, Sphingomonadales, Sphingomonadaceae | 30 | **-** | **+** | **+** | **-** | **-** | **-** | **-** | **+** |
| **L-1021** | *Sphingomonas paucimobilis* | Proteobacteria, Alphaproteobacteria, Sphingomonadales, Sphingomonadaceae | 29 | **+** | **+** | **+** | **-** | **+** | **-** | **-** | **+** |
| **L-969** | *Sphingomonas paucimobilis* | Proteobacteria, Alphaproteobacteria, Sphingomonadales, Sphingomonadaceae | 29 | **+** | **-** | **+** | **-** | **+** | **-** | **-** | **+** |
| **L-726** | *Staphylococcus pasteuri* | Firmicutes, Bacilli, Bacillales, Staphylococcaceae | 10 | **-** | **-** | **-** | **-** | **-** | **-** | **-** | **+** |
| **L-761** | *Staphylococcus saprophyticus* | Firmicutes, Bacilli, Bacillales, Staphylococcaceae | 15 | **-** | **+** | **-** | **-** | **-** | **-** | **-** | **+** |
| **L-899** | *Staphylococcus saprophyticus* | Firmicutes, Bacilli, Bacillales, Staphylococcaceae | 25 | **+** | **+** | **+** | **-** | **+** | **-** | **-** | **+** |
| **L-752** | *Staphylococcus saprophyticus* | Firmicutes, Bacilli, Bacillales, Staphylococcaceae | 29 | **+** | **+** | **-** | **-** | **-** | **-** | **-** | **+** |
| **L-753** | *Staphylococcus saprophyticus* | Firmicutes, Bacilli, Bacillales, Staphylococcaceae | 29 | **+** | **+** | **-** | **-** | **-** | **-** | **-** | **+** |
| **L-571** | *Staphylococcus saprophyticus* | Firmicutes, Bacilli, Bacillales, Staphylococcaceae | 30 | **+** | **+** | **-** | **-** | **+** | **-** | **-** | **+** |
| **L-572** | *Staphylococcus* sp. | Firmicutes, Bacilli, Bacillales, Staphylococcaceae | 29 | **+** | **+** | **+** | **-** | **+** | **-** | **-** | **+** |
| **L-382** | *Staphylococcus* sp. | Firmicutes, Bacilli, Bacillales, Staphylococcaceae | 4 | **+** | **+** |  | **-** | **+** | **-** | **-** | **+** |
| **L-717** | *Staphylococcus* sp. | Firmicutes, Bacilli, Bacillales, Staphylococcaceae | 6 | **+** | **+** | **-** | **-** | **+** | **-** | **-** | **+** |
| **L-1104** | *Staphylococcus succinus* | Firmicutes, Bacilli, Bacillales, Staphylococcaceae | 28 | **-** | **+** | **-** | **-** | **+** | **-** | **-** | **+** |
| **L-655** | *Stenotrophomonas maltophilia* | Proteobacteria, Gammaproteobacteria, Xanthomonadales, Xanthomonadaceae | 1 | **+** | **+** | **+** | **+** | **+** | **-** | **+** | **+** |
| **L-656** | *Stenotrophomonas maltophilia* | Proteobacteria, Gammaproteobacteria, Xanthomonadales, Xanthomonadaceae | 1 | **+** | **+** | **+** | **+** | **+** | **-** | **+** | **+** |
| **L-665** | *Stenotrophomonas maltophilia* | Proteobacteria, Gammaproteobacteria, Xanthomonadales, Xanthomonadaceae | 1 | **+** | **+** | **+** | **+** | **+** | **-** | **+** | **+** |
| **L-575** | *Stenotrophomonas maltophilia* | Proteobacteria, Gammaproteobacteria, Xanthomonadales, Xanthomonadaceae | 10 | **+** | **+** | **-** | **-** | **-** | **-** | **-** | **+** |
| **L-788** | *Stenotrophomonas maltophilia* | Proteobacteria, Gammaproteobacteria, Xanthomonadales, Xanthomonadaceae | 10 | **+** | **+** | **+** | **+** | **+** | **-** | **+** | **+** |
| **L-812** | *Stenotrophomonas maltophilia* | Proteobacteria, Gammaproteobacteria, Xanthomonadales, Xanthomonadaceae | 10 | **+** | **+** | **+** | **+** | **+** | **+** | **+** | **+** |
| **L-981** | *Stenotrophomonas maltophilia* | Proteobacteria, Gammaproteobacteria, Xanthomonadales, Xanthomonadaceae | 10 | **+** | **+** | **+** | **+** | **+** | **-** | **+** | **+** |
| **L-635** | *Stenotrophomonas maltophilia* | Proteobacteria, Gammaproteobacteria, Xanthomonadales, Xanthomonadaceae | 2 | **+** | **+** | **+** | **+** | **+** | **-** | **-** | **+** |
| **L-699** | *Stenotrophomonas maltophilia* | Proteobacteria, Gammaproteobacteria, Xanthomonadales, Xanthomonadaceae | 2 | **+** | **+** | **+** | **+** | **+** | **-** | **-** | **+** |
| **L-961** | *Stenotrophomonas maltophilia* | Proteobacteria, Gammaproteobacteria, Xanthomonadales, Xanthomonadaceae | 2 | **+** | **+** | **+** | **+** | **+** | **-** | **-** | **+** |
| **L-945** | *Stenotrophomonas maltophilia* | Proteobacteria, Gammaproteobacteria, Xanthomonadales, Xanthomonadaceae | 20 | **+** | **+** | **+** | **-** | **+** | **-** | **+** | **+** |
| **L-574** | *Stenotrophomonas maltophilia* | Proteobacteria, Gammaproteobacteria, Xanthomonadales, Xanthomonadaceae | 23 | **+** | **-** | **+** | **+** | **+** | **+** | **+** | **+** |
| **L-692** | *Stenotrophomonas maltophilia* | Proteobacteria, Gammaproteobacteria, Xanthomonadales, Xanthomonadaceae | 23 | **+** | **+** | **+** | **+** | **+** | **-** | **-** | **+** |
| **L-860** | *Stenotrophomonas maltophilia* | Proteobacteria, Gammaproteobacteria, Xanthomonadales, Xanthomonadaceae | 23 | **+** | **-** | **+** | **+** | **+** | **-** | **-** | **+** |
| **L-1064** | *Stenotrophomonas maltophilia* | Proteobacteria, Gammaproteobacteria, Xanthomonadales, Xanthomonadaceae | 23 | **+** | **-** | **+** | **+** | **+** | **-** | **+** | **+** |
| **L-573** | *Stenotrophomonas maltophilia* | Proteobacteria, Gammaproteobacteria, Xanthomonadales, Xanthomonadaceae | 24 | **+** | **+** | **+** | **+** | **+** | **-** | **+** | **+** |
| **L-681** | *Stenotrophomonas maltophilia* | Proteobacteria, Gammaproteobacteria, Xanthomonadales, Xanthomonadaceae | 24 | **+** | **+** | **+** | **+** | **+** | **-** | **+** | **+** |
| **L-857** | *Stenotrophomonas maltophilia* | Proteobacteria, Gammaproteobacteria, Xanthomonadales, Xanthomonadaceae | 24 | **+** | **-** | **+** | **+** | **+** | **+** | **+** | **+** |
| **L-937** | *Stenotrophomonas maltophilia* | Proteobacteria, Gammaproteobacteria, Xanthomonadales, Xanthomonadaceae | 25 | **+** | **+** | **+** | **+** | **+** | **-** | **+** | **+** |
| **L-702** | *Stenotrophomonas maltophilia* | Proteobacteria, Gammaproteobacteria, Xanthomonadales, Xanthomonadaceae | 25 | **+** | **-** | **+** | **+** | **+** | **-** | **+** | **+** |
| **L-1059** | *Stenotrophomonas maltophilia* | Proteobacteria, Gammaproteobacteria, Xanthomonadales, Xanthomonadaceae | 25 | **+** | **+** | **+** | **+** | **+** | **+** | **+** | **+** |
| **L-1070** | *Stenotrophomonas maltophilia* | Proteobacteria, Gammaproteobacteria, Xanthomonadales, Xanthomonadaceae | 25 | **+** | **-** | **+** | **+** | **+** | **-** | **+** | **+** |
| **L-787** | *Stenotrophomonas maltophilia* | Proteobacteria, Gammaproteobacteria, Xanthomonadales, Xanthomonadaceae | 5 | **+** | **+** | **+** | **+** | **+** | **-** | **+** | **+** |
| **L-412** | *Stenotrophomonas maltophilia* | Proteobacteria, Gammaproteobacteria, Xanthomonadales, Xanthomonadaceae | 6 | **+** | **+** | **+** | **+** | **+** | **-** | **+** | **+** |
| **L-757** | *Stenotrophomonas maltophilia* | Proteobacteria, Gammaproteobacteria, Xanthomonadales, Xanthomonadaceae | 8 | **+** | **+** | **+** | **+** | **+** | **+** | **+** | **+** |
